# Identification of β Clamp-DNA Interaction Regions That Impair the Ability of *E*. *coli* to Tolerate Specific Classes of DNA Damage

**DOI:** 10.1371/journal.pone.0163643

**Published:** 2016-09-29

**Authors:** Michael T. Nanfara, Vignesh M. P. Babu, Mohamed A. Ghazy, Mark D. Sutton

**Affiliations:** 1 Department of Biochemistry, Jacobs School of Medicine and Biomedical Sciences, University at Buffalo, State University of New York, Buffalo, NY, 14214, United States of America; 2 Department of Biochemistry, Faculty of Science, Ain Shams University, Cairo, 11566, Egypt; Cornell University, UNITED STATES

## Abstract

The *E*. *coli dnaN*-encoded β sliding clamp protein plays a pivotal role in managing the actions on DNA of the 5 bacterial DNA polymerases, proteins involved in mismatch repair, as well as several additional proteins involved in DNA replication. Results of *in vitro* experiments indicate that the loading of β clamp onto DNA relies on both the DnaX clamp loader complex as well as several discrete sliding clamp-DNA interactions. However, the importance of these DNA interactions to *E*. *coli* viability, as well as the ability of the β clamp to support the actions of its numerous partner proteins, have not yet been examined. To determine the contribution of β clamp-DNA interactions to the ability of *E*. *coli* to cope with different classes of DNA damage, we used alanine scanning to mutate 22 separate residues mapping to 3 distinct β clamp surfaces known or nearby those known to contact the DNA template, including residues P20-L27 (referred to here as loop I), H148-Y154 (loop II) and 7 different residues lining the central pore of the β clamp through which the DNA template threads. Twenty of these 22 *dnaN* mutants supported bacterial growth. While none of these 20 conferred sensitivity to hydrogen peroxide or ultra violet light, 12 were sensitized to NFZ, 5 were sensitized to MMS, 8 displayed modestly altered frequencies of DNA damage-induced mutagenesis, and 2 may be impaired for supporting *hda* function. Taken together, these results demonstrate that discrete β clamp-DNA interaction regions contribute to the ability of *E*. *coli* to tolerate specific classes of DNA damage.

## Introduction

The DnaN/β family of sliding clamp proteins plays important roles in coordinating the actions on DNA of a diverse set of proteins involved in DNA replication, repair and translesion synthesis (TLS) (reviewed in [1–3]). To fulfill this role, the clamp must topologically encircle DNA. Clamps are assembled onto DNA by multimeric ATPases called clamp loaders (reviewed in [4,5]). The *E*. *coli* DnaX clamp loader complex (comprised of τ_2_γδδ’ψχ) pries open the *dnaN*-encoded homodimeric β clamp at one of its two interfaces in an ATP-dependent manner [6–9]. DnaX-ATP_3_ in complex with the open β clamp binds to double strand (ds)/single strand (ss) DNA junctions specifically recognizing the 3’-OH end of the ssDNA [10]. The DnaX clamp loader hydrolyzes bound ATP effecting release of both the clamp and DNA, allowing the β homodimer to close around dsDNA [11]. Once “loaded” onto dsDNA, the β clamp slides freely and recruits its various partner proteins, including each of the five *E*. *coli* DNA polymerases (Pols I-V) [12–26]. However, the mechanisms underlying the ability of β clamp to coordinately regulate the actions on DNA of its different partner proteins remain poorly understood (reviewed in [1]).

In addition to DnaX clamp loader, β clamp-DNA interactions also contribute to the loading mechanism [5,27,28]. At least 4 distinct surfaces of the β clamp physically contact the DNA template in a nucleotide sequence-independent manner, including specific residues located within two distinct loops, referred to in this study as loop I (residues P20-L27) and loop II (H148-Y154), a hydrophobic cleft that is known to interact with most if not all of the clamp partner proteins via a conserved clamp binding motif [29], and several residues lining the central pore of the clamp. Position R24 in loop I, together with Q149, Y153 and Y154 in loop II, which contact DNA, contribute to loading of β clamp onto DNA by the DnaX complex *in vitro*: contributions to loading of other β clamp residues known to contact the DNA template were not investigated [27]. Once loaded, however, these residues were dispensable for Pol III replicase function *in vitro* [27,28]. A mutant β clamp bearing alanine substitutions of H148-R152 (β^148–152^) was unable to support *E*. *coli* growth, consistent with loop II-DNA interactions serving an essential role in one or more β clamp functions [28]. However, Pol IV (as well as Pol II) interacts with loop II of the β clamp, and this interaction is required for processive replication *in vitro* [28]. Moreover, physiological levels of β^148–152^ expressed from a low-copy-number plasmid complemented the temperature sensitive growth phenotype of the *dnaN159*(Ts) strain provided the lesion bypass *dinB*-encoded Pol IV was inactivated, suggesting loop II in β clamp contributes to proper coordination of replication and TLS [28,30]. Thus, the contribution of β clamp-DNA interactions to TLS is currently unclear.

In addition to managing the actions of the 5 *E*. *coli* Pols to coordinate high fidelity replication with potentially mutagenic TLS, the β clamp also participates in at least 3 additional cellular functions. First, interaction of the β clamp with MutS and MutL contributes to mismatch repair *in vivo*, ensuring that DNA is replicated faithfully [31,32]. Second, CrfC, a dynamin homolog, exploits its ability to interact with multiple β clamps that accumulate on lagging strand following DNA replication to appropriately position sister replication forks for subsequent chromosome partitioning [33]. Finally, the β clamp interacts with Hda to regulate its ability to dramatically stimulate the ATPase activity of the DnaA replication initiator protein via a process termed regulatory inactivation of DnaA, or RIDA [34]. While the ATP-bound form of DnaA (DnaA-ATP) is active for initiation of DNA replication, the ADP-bound form (DnaA-ADP) is not. Thus, Hda, together with the β clamp, act to regulate initiation by controlling the level of active DnaA in the cell.

**Fig 1:**
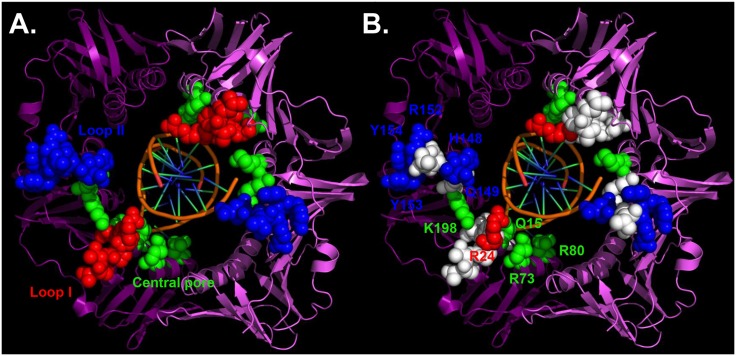
Relative positions of mutations examined in this study. **(A)** Residues in the β clamp analyzed in this study comprising loop I (P20-L27 in red) and loop II (H148-Y154 in blue), or mapping to the central pore of the β clamp (K12, Q15, Q16, R73, R80, R197 & K198 in green) are highlighted as colored space filled atoms. **(B)** Positions of residues mutated within these regions that are involved in direct interaction with the DNA template as defined by Georgescu and coworkers [27] are colored as in panel A, while those that do not make direct contact with DNA are colored white. Interaction of residue R152, Y153 and Y154 with the DNA were mediated by the symmetry-related β molecule [27]. This image was generated using the MacPyMol Molecular Graphics System, Ver. 1.7.4 Schrodinger, LLC and the coordinates for the crystal structure of the β clamp-DNA complex (PBD: 3BEP).

The goal of this work was to determine whether residues in the β sliding clamp that directly contact the DNA template contribute to *E*. *coli* viability and/or its ability to effectively cope with DNA damage by TLS. To this end, we characterized a total of 22 *dnaN* mutations targeting positions in loop I, loop II and the central pore of the β clamp. Ten of these 22 mutations specifically targeted residues known to contact the DNA template (Fig 1). The other 12 mutations targeted adjacent residues that did not contact the DNA template in the β clamp-DNA complex, but might in solution. Using a shuffle assay that exploits a Δ*dnaN*::(*kan*, *sacB*) allele, we analyzed phenotypes of these mutant β clamps when expressed at physiological levels from a low-copy-number plasmid. Twenty of the 22 *dnaN* mutations supported *E*. *coli* viability. Although loop I, loop II and the central pore were each vitally important for protecting *E*. *coli* against killing by nitrofurazone (NFZ), only loop I and the central pore were required for protection from methyl methanesulfonate (MMS). In contrast, these three regions of the β clamp were not required for protection from killing by ultraviolet light (UV) or hydrogen peroxide (H_2_O_2_). Finally, 8 *dnaN* mutants displayed modestly altered frequencies of DNA damage induced mutagenesis, while 2 others appear to be impaired for supporting *hda* function. Taken together, these results demonstrate that discrete β clamp-DNA interaction regions contribute to the ability of *E*. *coli* to tolerate specific classes of DNA damage.

## Materials and Methods

### Bacteriological techniques

Bacterial strains and plasmid DNAs used in this study are described in S1 Table; oligonucleotides are described in S2 Table. Strains were constructed using λ recombineering [35], generalized P1*vir* transduction [36], and CaCl_2_-mediated transformation [16], as described in the indicated references. Strains were grown in Luria-Bertani (LB: 10 g/l Difco tryptone, 5 g/l Difco yeast extract, 10 g/l NaCl), or M9 minimal medium (M9: 12.9 g/l Na_2_HPO_4_•7H_2_O, 3 g/l KH_2_PO_4_, 0.5 g/l NaCl, 1 g/l NH_4_Cl) supplemented with 0.1 mM CaCl_2_, 2 mM MgCl_2_, 40 μg/ml thiamine and 0.5% maltose, as indicated. When appropriate, growth medium was supplemented either with 15 g/l (agar plates) or 7.5 g/l (top agar) Difco agar, and/or 150 μg/ml ampicillin (Amp), 20 μg/ml kanamycin (Kan), 20 μg/ml chloramphenicol (Cam) and/or 50 μg/ml rifampicin (Rif).

### Construction of the Δ*dnaN*::(*kan*, *sacB*) strain

The genetic structure of the Δ*dnaN*::(*kan*, *sacB*) allele, summarized in S1 Fig, was constructed as follows: primers DnaA-BamHI-Fwd and DnaA-NheI-Rev, KanR-NheI-Fwd and KanR-XhoI-Rev, SacB-XhoI-Fwd and SacB-NotI-Rev, and RecF-NotI-Fwd and RecF-XbaI-Rev (S2 Table) were used to PCR amplify fragments corresponding to the 3’-most 762 bp of *dnaA*, the 945 bp *kan* gene, the 1,714 bp *sacB* allele, and the 5’-most 700 bp of *recF*. These fragments were individually cloned into the Zero Blunt TOPO vector (ThermoFisher) then sequentially joined at the unique *NheI* (*dnaA–kan*), *XhoI* (*kan–sacB*) and *NotI* (*sacB–recF*) sites by standard ligation to Zero Blunt TOPO, ultimately generating plasmid pAKSF-PCR TOPO containing the *dnaN*::(*kan*, *sacB*) locus flanked by *dnaA* and *recF* sequences for recombination with the chromosomal *dnaN* locus. The 4.1 kbp ‘*dnaA*–Δ*dnaN*::(*kan*, *sacB*)–*recF’* fragment was PCR amplified from plasmid pAKSF-PCR TOPO using primers DnaA-BamHI-Fwd and RecF-XbaI-Rev (S2 Table), gel purified, and electroporated into strain MO90-3 as described [28,37], resulting in strain MO90-3-AKSF (S1 Table). Representative transductants, selected on M9 supplemented with maltose and Kan, were confirmed to contain the Δ*dnaN*::(*kan*, *sacB*) allele in place of the normal *dnaN*^*+*^ locus using diagnostic PCR as described [28,37]; growth of these clones was dependent on the *lamB*::(His_6_-*dnaN*^*+*^–*cam*) allele. Representative MO90-3-AKSF clones were further confirmed by nucleotide sequence analysis (Roswell Park Biopolymer Facility, Buffalo, NY) of the PCR amplified Δ*dnaN*::(*kan*, *sacB*) allele from genomic DNA using primers DnaAP and RecF-Bottom.

### Cloning mutant *dnaN* plasmids

Plasmids expressing mutant *dnaN* alleles were constructed using the QuickChange kit (ThermoFisher Scientific). Oligonucleotides used to generate each mutant are described in S2 Table. Each mutant *dnaN* allele was sequenced completely (Roswell Park Biopolymer Facility, Buffalo, NY) using primers Beta For1 SP and Beta Rev1 SP.

### Measure of the ability of plasmid-expressed *dnaN* alleles to support viability

The ability of each plasmid-expressed *dnaN* mutant to support *E*. *coli* growth was measured using a shuffle assay. *E*. *coli* strain MG1655 was transformed with the indicated β clamp-expressing plasmid. Two independent transformants corresponding to each *dnaN* mutant were transduced to Kan^R^ using P1*vir* grown on strain MO90-3-AKSF (relevant genotype: Δ*dnaN*::(*kan*, *sacB*)). As a control, select strains were also transduced with P1*vir* grown on strain MN000 (relevant genotype: *tnaA300*::Tn10*kan dnaN*^*+*^). Transduction frequency was calculated by dividing the number of Kan^R^ CFU by the number of P1*vir* PFU used in the transduction.

### Measurement of steady state levels of mutant β clamp proteins

Western blotting was performed essentially as described previously [30]. Briefly, overnight cultures of the indicated strains were sub-cultured to mid-exponential phase, and 5 x 10^8^ cells of each strain were collected by centrifugation. Cell pellets were resuspended in 100 μl 2X SDS-PAGE loading buffer (100 mM Tris-HCl (pH 6.8), 4% SDS, 0.1% bromophenyl blue, 20% glycerol, 5% mercaptoethanol). Ten-μl of each whole cell lysate was electrophoresed in 12% SDS-PAGE then transferred to polyvinylidene difluoride (PVDF) using a Trans Blot Turbo semi-dry transfer apparatus (Bio-Rad). After transfer, PVDF membranes were washed with TS Buffer (20 mM Tris-HCl (pH 7.6) and 150 mM NaCl) then blocked with TS Buffer containing 0.1% Tween 20 and 2% non-fat dry milk. Washed membranes were probed with rabbit polyclonal anti-β clamp (1:20,000 dilution) antibodies [30]. After washing, PVDF membranes were probed with goat anti-rabbit antibody (1:50,000) and β clamp was detected using the Clarity Western ECL Chemiluminescence substrate (Bio-Rad). Quantitation was performed using a ChemiDoc Imager equipped with the Image Lab software (Bio-Rad). Serial dilutions of wild type extracts were used to verify that observed levels of β clamp were within the linear range of detection.

### Measurement of mutant *dnaN* strain growth rates

Overnight cultures were sub-cultured 1:200 into 200 μl of LB in quadruplicate in sterile 96 well microtiter plates. Plates were incubated at 37°C with shaking, and growth was monitored by measuring the change in optical density at 600 nm (OD_600 nm_) every 20 min using a FLUOstar Omega microplate reader (Ingen Technologies). The doubling time for each strain was calculated using the doubling time cell calculator (Roth V. 2006 Doubling Time Computing, available from: http://www.doubling-time.com/compute.php).

### Susceptibility of mutant *dnaN* strains to DNA damaging agents

Sensitivity of wild type and mutant *dnaN* strains to methyl methanesulfonate (MMS) [37], nitrofurazone (NFZ) [38], ultraviolet light (UV) [23,37], or hydrogen peroxide (H_2_O_2_) [39] was measured essentially as described in the indicated references. Briefly, sensitivity to MMS and NFZ was measured by spotting 10 μl aliquots of 10-fold serial dilutions of exponential cultures onto LB agar plates supplemented with the indicated concentrations of drug followed by overnight incubation at 37°C. NFZ was suspended in dimethylformamide (DMF), and for the 0 NFZ control, plates were supplemented with DMF alone. For UV, 10-fold serial dilutions of exponential cultures were spotted onto LB agar plates. Spots were allowed to dry then plates were exposed to UV (254 nm) using a 15-W germicidal bulb (General Electric) prior to overnight incubation at 37°C. Sensitivity to H_2_O_2_ was measured using a disk diffusion assay [39]. One hundred-μl of overnight culture was mixed with 3 ml of molten LB top agar and overlaid on top of an LB agar plate supplemented with Amp. After the top agar solidified (30 min), a sterile 6 mm circular Whatman filter was placed on top of the plate. Ten-μl of either 3% H_2_O_2_ (Fisher Scientific) or sterile water (as a negative control) was pipetted onto the filter, and the diameter of the zone of clearance was measured after overnight incubation at 37°C.

### Ability of mutant *dnaN* strains to support DNA damage-induced mutagenesis

MMS-induced mutagenesis was measured by inoculating 5 ml of LB supplemented with 0.5–2.0 mM (for *dinB*^*+*^ strains), as indicated, or 1 mM MMS (for Δ*dinB* strains), with 100 μl of exponential cells (OD_600 nm_ = 0.5). Cultures were grown overnight at 37°C. UV-induced mutagenesis was measured using exponential cells (OD_600 nm_ = 0.5) that were washed and resuspended in 0.8% saline. Five hundred-μl of UV (254 nm) irradiated cells (60 J/m^2^) were inoculated into 4.5 ml of LB and cultured overnight at 37°C. Appropriate dilutions of both MMS and UV treated cells were plated onto either LB agar plates (cell titer) or LB agar supplemented with 50 μg/ml Rif (mutants), and frequencies of MMS- and UV-induced mutagenesis (Rif^R^) were calculated as described previously [30].

### Measurement of *oriC*/*terC* ratios

*oriC* and *terC* copy numbers were measured using quantitative PCR (qPCR) as described [40]. Briefly, cultures were grown at 37°C in LB medium to an OD_600nm_ = 0.5–0.8. As a control, we used a freshly constructed Δ*hda*::*cat* strain that was unable to growth at 30°C, indicating it was unsuppressed [40]. The genomic DNA was extracted from 1 ml of each cell culture using the Sigma^™^ GenElute^™^ Bacterial Genomic DNA kit as per the manufacturer’s recommendations. Quantitative PCR was performed using Bio-Rad SsoAdvanced^™^ Universal SYBR^®^ Green Supermix in Bio-Rad CFX96 Connect Real-Time PCR Detection System with manual Cq threshold of 60. PCR reactions (20 μl) contained 10 μl of 2x SYBR Green Supermix, 1.5 μl of genomic DNA (0.3 ng/μl) and 250 nM of each of the oriC-1 and oriC-2 primers, or terC-1 and terC-2 primers, which amplify 150 bp fragments near the origin or terminus, respectively. PCR reactions were heated 98°C for 3 min, followed by 40 cycles with steps of 98°C, 56°C and 72°C for 30s each. The generation of specific PCR products was confirmed using melting curve analysis.

### Statistical analysis

Statistical significance for spontaneous mutation was based on 95% confidence intervals (CI) [41]. For all other results, statistical significance was determined using the Evan Miller t-test tool (http://www.evanmiller.org/ab-testing/t-test.html).

## Results

### Objectives of this study and motivation for the design of *dnaN* mutations

*E*. *coli* β clamp-DNA interactions contribute to the loading of β clamp onto DNA by the DnaX clamp loader complex *in vitro* [27,28]. However, a comprehensive analysis of the contributions of the different β clamp-DNA interactions to *in vivo* clamp function has not yet been described. In this work, we used a novel shuffle assay to measure the respective abilities of mutant β clamp proteins to support *E*. *coli* viability and to cope with DNA damage by TLS *in vivo*. Using the β clamp-DNA crystal structure as a guide [27], we performed alanine scanning mutagenesis of three of the four regions in the β clamp demonstrated to contact the DNA template, including loop I (residues P20-L27), loop II (residues H148-Y154) and the inner lining of the β clamp central pore through which dsDNA threads (Fig 1). We targeted residues in the β clamp previously demonstrated to contact DNA, as well as several residues adjacent to these that may also contact DNA under conditions that differ from those used for crystallization [27]. Based on results of *in vitro* experiments, single mutants of β clamp targeting residues known to contact DNA were still loaded onto DNA fairly efficiently, while loading of double mutants was severely impaired [27]. Therefore, to limit the possibility that a *dnaN* mutant phenotype was the result of a failure to load that β clamp protein onto DNA, we analyzed phenotypes of single amino acid substitution mutations. We did not examine the hydrophobic cleft, which interacts with ssDNA, due to the fact that this β clamp surface also interacts with the β clamp-binding motif present in most if not all clamp-binding partners, making this region of the β clamp essential for bacterial viability [29,42–44]. Since loop II also interacts with Pol II and Pol IV [28], we decided to substitute each residue in loop I and loop II individually. We hypothesized that if disruption of a particular β clamp-DNA interaction was responsible for an observed mutant phenotype, then mutations targeting nearby residues not involved in DNA interactions should lack this phenotype. Alternatively, if the phenotype was the result of an altered partner protein interaction, then substitution of nearby residues that based on the crystal structure failed to contact the DNA template should confer the mutant phenotype. In addition to alanine scanning, we also substituted with glutamic acid two separate β clamp residues lining the central pore of the clamp. We chose a K-to-E substitution hypothesizing that a charge reversal at either of these positions might alter the way in which the β clamp sits on DNA to confer a more pronounced phenotype than would an alanine substitution. One of these residues (K198) directly contacts DNA in the β clamp-DNA crystal, while the other (K12) failed to make contact, but is immediately adjacent to other residues that did contact the DNA template [27]. Furthermore, residues K12 and K198 map to opposite ends of a basic patch lining the inner pore of the β clamp channel that, together with residues in loops I and II, make subunit-specific contacts with the DNA template, holding it at a ~22° angle relative to the horizontal axis of the central pore of the β clamp ([27]; see Fig 1). Below we describe results of experiments aimed at measuring the respective abilities of these mutant β clamps to support *E*. *coli* viability and DNA damage tolerance *in vivo*. Finally, the level of amino acid conservation (%) at each *dnaN* position mutated in this study based on an alignment of deduced β clamp protein sequences of 90 distinct bacterial species is indicated in Table 1.

**Table 1:** Summary of mutant *dnaN* phenotypes ^a^.

Mutant class	*dnaN* mutation	Conservation (%): ^b^		Known DNA contact	Viability	Relative growth rate	Sensitivity to:		Mutagenesis:			Relative *oriC*/*terC* ratio
		Identity	Similarity				NFZ	MMS	Spontaneous	UV	MMS	
**Control**	WT	*na*	*na*	*na*	+	+	+	+	+	+	+	≡1.0
**Loop I**	P20A	10	10	–	+	F	S	+	+	+	+	1.4
	L21A	26	97	–	+	+	+	S	+	+	+	1.3
	G22A	16	39	–	+	+	+	+	+	+	2x↑	*nd*
	G23A	18	27	–	+/–	F	S	+	+	+	+	1.1
	R24A	61	91	+	+/–	F	S	+	+	+	3x↑	1.5
	P25A	24	29	*–*	+	+	+	+	+	+	+	*nd*
	T26A	66	74	–	+	+	S	S	+	+	+	0.9
	L27A	41	84	–	+	+	+	+	+	+	+	*nd*
**Loop II**	H148A	11	26	+	+	+	+	+	+	+	+	*nd*
	Q149A	42	50	+	+	+	+	+	2x↑	2x↑	+	*nd*
	D150A	26	94	–	–	+	*na*	*na*	+	*na*	*na*	*nd*
	V151A	27	43	–	+/–	+	HS	+	+	2x↓	+	1.7
	R152A	82	89	+^c^	–	+	*na*	*na*	+	*na*	*na*	*nd*
	Y153A	50	52	+ ^c^	+	F	S	+	+	+	+	1.1
	Y154A	40	46	+ ^c^	+	+	+	+	+	+	+	*nd*
**Central pore**	K12E	46	62	–	+	+	HS	S	2x↓	+	>10x↓	1.1
	Q15A	40	72	+	+	+	+	+	+	+	+	*nd*
	Q16A	18	32	–	+	F	S	+	+	+	+	0.8
	R73A	51	82	+	+	+	HS	+	+	2x↑	+	2.9
	R80A	53	87	+	+	F	HS	S	+	+	2x↓	1.0
	R197A	46	51	–	+	F	HS	S	+	+	3x↓	1.1
	K198E	87	98	+	+	+	HS	+	2x↓	+	+	1.0

^***a***^ Symbols are as follows: WT, wild type; +, yes, proficient or wild type phenotype; –, no or deficient; +/–, proficient but modestly impaired; F, growth is faster than WT; S, sensitive compared to wild type; HS, hypersensitive compared to wild type; 2x↑, 2-fold higher mutations frequency than wild type; 3x↑, 3-fold higher than wild type; 2x↓, 2-fold lower than wild type; 3x↓, 3-fold lower than wild type; >10↓, more than 10-fold lower than wild type; *na*, not applicable; *nd*, not determined.

^***b***^ Conservation (identity or similarity) of each amino acid position is based on an alignment of 90 different bacterial species whose genomes were fully sequenced. The alignment was generated using Blink (http://www.ncbi.nlm.nih.gov/sutils/blink.cgi?mode=query).

^***c***^ Interaction of these β clamp residues with the DNA template involved the symmetry-related β molecule [27]. However, the ability of Y153 and Y154 to interact with DNA, and the functional importance of these interactions to loading β clamp onto DNA *in vitro* was confirmed.

### Residues D150 and R152 in loop II of the β clamp are required for *E*. *coli* viability

Using a *dnaN* shuffle assay, we asked whether physiological levels of site-specific mutant β clamp proteins expressed from a low-copy-number plasmid supported *E*. *coli* growth. For this assay, we first transformed *E*. *coli* strain MG1655 with the empty cloning plasmid pWSK29, or pWSK29 derivatives expressing either wild type β clamp (pJD100) or one of the mutant clamp proteins (pMN plasmids; see S1 Table). We then quantitatively measured the ability of each mutant *dnaN* allele to support bacterial growth after replacing the native chromosomal *dnaN*^*+*^ allele with Δ*dnaN*::(*kan*, *sacB*) from strain MO90-3-AKSF using generalized P1*vir* transduction. As summarized in Table 2, the wild type-expressing plasmid pJD100 supported efficient replacement of the chromosomal *dnaN*^*+*^ allele with Δ*dnaN*::(*kan*, *sacB*) as measured by transduction efficiency to Kan^R^ (3.5±0.5 x 10^−4^ Kan^R^ CFU/PFU). In contrast, the empty control plasmid (pWSK29) failed to yield Kan^R^ transductants (<2.0 x 10^−7^ Kan^R^ CFU/PFU). In order to confirm the pWSK29 strain was both susceptible to P1*vir* infection and capable of recombination, we used a P1*vir* lysate grown on the *tnaA300*::Tn*10kan* strain MN000: the *tnaA* allele is ~80% linked to *dnaN*^*+*^ [45]. As summarized in Table 2, the pJD100 and pWSK29 strains displayed comparable efficiencies of Kan^R^ transduction with *tnaA300*::Tn*10kan*, confirming that this shuffle assay accurately measures the ability of mutant *dnaN* alleles to support *E*. *coli* viability, similar to a previously described system [46].

**Table 2:** Abilities of mutant *dnaN* alleles to support *E*. *coli* viability.

Mutant class	Plasmid	*dnaN* allele	P1*vir* transduction efficiency (Kan^R^ CFU/P1*vir* PFU) for: ^a^	
			Δ*dnaN*::(*kan*, *sacB*)	*dnaN*^*+*^ *tnaA300*::Tn*10kan*
**Controls**	pJD100	*dnaN*^*+*^	3.5 (±0.5) x 10^−4^	2.3 (±0.5) x 10^−4^
	pWSK29	none	< 2.0 x 10^−7^	2.5 (±0.1) x 10^−4^
**Loop I**	pMN100	*dnaN-P20A*	0.7 (±0.4) x 10^−4^	*nd*
	pMN101	*dnaN-L21A*	3.8 (±1.3) x 10^−4^	*nd*
	pMN102	*dnaN-G22A*	2.3 (±0.2) x 10^−4^	*nd*
	pMN103 ^b^	*dnaN-G23A*	0.2 (±0.1) x 10^−4^	3.5 (±1.9) x 10^−4^
	pMN104 ^b^	*dnaN-R24A*	0.2 (±0.1) x 10^−4^	1.1 (±1.0) x 10^−4^
	pMN105	*dnaN-P25A*	3.1 (±0.4) x 10^−4^	*nd*
	pMN106	*dnaN-T26A*	0.7 (±0.1) x 10^−4^	*nd*
	pMN107	*dnaN-L27A*	3.4 (±1.8) x 10^−4^	*nd*
**Loop II**	pMN108	*dnaN-H148A*	2.5 (±0.5) x 10^−4^	*nd*
	pMN109	*dnaN-Q149A*	1.6 (±0.9) x 10^−4^	*nd*
	pMN110	*dnaN-D150A*	< 2.0 x 10^−7^	3.8 (±1.8) x 10^−4^
	pMN111 ^b^	*dnaN-V151A*	0.2 (±0.1) x 10^−4^	2.8 (±1.1) x 10^−4^
	pMN112	*dnaN-R152A*	< 2.0 x 10^−7^	3.5 (±1.4) x 10^−4^
	pMN113	*dnaN-Y153A*	0.8 (±0.3) x 10^−4^	*nd*
	pMN114	*dnaN-Y154A*	2.1 (±0.2) x 10^−4^	*nd*
**Central pore**	pMN115	*dnaN-K12E*	1.4 (±0.6) x 10^−4^	*nd*
	pMN116	*dnaN-Q15A*	2.3 (±0.1) x 10^−4^	*nd*
	pMN117	*dnaN-Q16A*	0.6 (±0.1) x 10^−4^	*nd*
	pMN118	*dnaN-R73A*	1.4 (±0.5) x 10^−4^	*nd*
	pMN119	*dnaN-R80A*	2.2 (±0.1) x 10^−4^	*nd*
	pMN120	*dnaN-R197A*	1.8 (±0.4) x 10^−4^	*nd*
	pMN121	*dnaN-K198E*	1.2 (±0.3) x 10^−4^	*nd*

^***a***^ Results represent the average of two separate determinations, each using an independent *dnaN* plasmid transformant, ± the range. *nd*, not determined.

^***b***^ The sequence of the indicated *dnaN* allele was confirmed in two independent transductants, confirming the lack of an intragenic suppressor mutation.

Using this shuffle assay, we demonstrated that 20 of the 22 mutant *dnaN* alleles were capable of supporting bacterial growth (Table 2). We were unable to construct the *dnaN-D150A* and *dnaN-R152A* shuffle strains. In contrast, these two mutant strains were transduced to Kan^R^ by *tnaA300*::Tn10*kan* with an efficiency comparable to the *dnaN*^*+*^ control strain (Table 2), supporting the conclusion that the *dnaN-D150A* and *dnaN-R152A* mutants were unable to support *E*. *coli* viability. Based on the β clamp-DNA crystal structure, D150 did not bind DNA, while residue R152 did. However, the interaction of R152 with DNA was observed in a symmetry-related clamp molecule [27]. Thus, the functional relevance of this DNA interaction is not yet confirmed.

Although viable, the *dnaN-G23A*, *dnaN-R24A* and *dnaN-V151A* strains were transduced ~10-fold less efficiently with Δ*dnaN*::(*kan*, *sacB*) compared to the *dnaN*^*+*^ control (Table 2), suggesting these mutants were partially deficient in one or more essential β clamp function. Nevertheless, these *dnaN* mutants were transduced with Δ*dnaN*::(*kan*, *sacB*) more than two orders of magnitude more efficiently than was the pWSK29 control. Moreover, this ~10-fold reduction in their respective transduction efficiencies was not attributable to a P1*vir* transduction defect, since these mutants were transduced to Kan^R^ with *tnaA300*::Tn10*kan* as efficiently as the *dnaN*^*+*^ control (Table 2). To rule out the possibility that these *dnaN* alleles acquired intragenic suppressor mutations, we isolated the plasmid from both independent isolates of each mutant and sequenced the *dnaN* allele in its entirety. None contained additional mutations. Thus, we conclude that *dnaN-G23A*, *dnaN-R24A* and *dnaN-V151A* are capable of supporting *E*. *coli* viability. Position R24 contacts the DNA template, while G23 and V151 are immediately adjacent to residues that contact DNA ([27]; see Fig 1).

**Fig 2:**
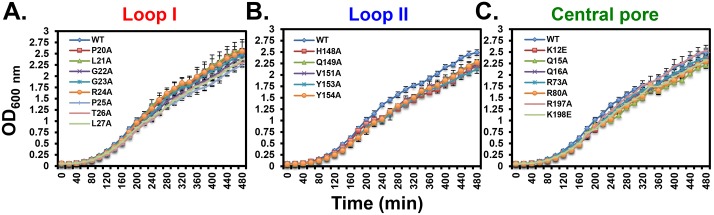
Ability of mutant *dnaN* alleles to support *E*. *coli* growth. Growth rates of *dnaN* strains bearing mutations in **(A)** loop I, **(B)** loop II or **(C)** the central pore of the β clamp were measured as described in *Materials and Methods*. Results represent an average of 4 determinations ± one standard deviation.

Based on quantitative Western blotting, each of the mutant β clamp proteins was expressed at levels comparable to the wild type control (Table 3). We also performed growth curves on the 20 viable shuffle strains to determine whether any of the mutant *dnaN* alleles impaired *E*. *coli* growth, paying particular attention to the *dnaN-G23A*, *dnaN-R24A* and *dnaN-V151A* strains (Fig 2). As summarized in Table 4, the *dnaN-G23A* and *dnaN-R24A* strains grew slightly faster (≤10%) than the wild type control, while the *dnaN-V151A* mutant was comparable to the wild type control. Five other mutants, including *dnaN-Q16A*, *dnaN-P20A*, *dnaN-R80A*, *dnaN-Y153A* and *dnaN-R197A* also grew slightly faster (≤15%) than wild type, while the rest were comparable to the wild type control. Taken together, findings discussed above demonstrate that with the exception of R152, *E*. *coli* viability is independent of the known β clamp-DNA interactions.

**Table 3:** Steady-state expression levels of mutant β clamp proteins.

Mutant class	*dnaN* allele	Relative expression level of β clamp ^a^
**Control**	*dnaN*^*+*^	≡1.0
**Loop I**	*dnaN-P20A*	1.40 (±0.24)
	*dnaN-L21A*	1.63 (±0.49)
	*dnaN-G22A*	1.25 (±0.27)
	*dnaN-G23A*	1.12 (±0.02)
	*dnaN-R24A*	1.23 (±0.04)
	*dnaN-P25A*	1.36 (±0.18)
	*dnaN-T26A*	1.21 (±0.01)
	*dnaN-L27A*	1.35 (±0.20)
**Loop II**	*dnaN-H148A*	1.27 (±0.26)
	*dnaN-Q149A*	1.12 (±0.32)
	*dnaN-V151A*	1.42 (±0.17)
	*dnaN-Y153A*	1.55 (±0.07)
	*dnaN-Y154A*	1.16 (±0.03)
**Central pore**	*dnaN-K12E*	0.94 (±0.03)
	*dnaN-Q15A*	0.75 (±0.15)
	*dnaN-Q16A*	0.99 (±0.39)
	*dnaN-R73A*	1.13 (±0.30)
	*dnaN-R80A*	1.03 (±0.15)
	*dnaN-R197A*	0.90 (±0.35)
	*dnaN-K198E*	1.83 (±0.30)

^***a***^ Respective levels of wild type and mutant β clamp proteins were determined by quantitative Western blotting. Results are expressed relative to β clamp levels observed in the wild type *dnaN*^*+*^ strain, which were set equal to 1.0 (≡1.0), and represent the average of two independent determinations, each involving s separate whole cell lysate, ± the range.

**Table 4:** Growth rates of mutant *dnaN* strains.

Mutant class	*dnaN* allele	Doubling time (min) ^a^
**Control**	*dnaN*^*+*^	47.5 (±1.5)
**Loop I**	*dnaN-P20A*	44.3 (±1.4) ^b^
	*dnaN-L21A*	47.7 (±1.5)
	*dnaN-G22A*	47.2 (±2.4)
	*dnaN-G23A*	44.7 (±0.7) ^b^
	*dnaN-R24A*	42.7 (±1.2) ^b^
	*dnaN-P25A*	47.4 (±2.3)
	*dnaN-T26A*	45.3 (±2.1)
	*dnaN-L27A*	48.8 (±2.7)
**Loop II**	*dnaN-H148A*	45.5 (±1.5)
	*dnaN-Q149A*	47.3 (±1.0)
	*dnaN-V151A*	44.9 (±2.4)
	*dnaN-Y153A*	42.8 (±1.5) ^b^
	*dnaN-Y154A*	45.6 (±2.3)
**Central pore**	*dnaN-K12E*	47.8 (±1.4)
	*dnaN-Q15A*	42.4 (±3.6)
	*dnaN-Q16A*	40.7 (±3.6) ^b^
	*dnaN-R73A*	49.7 (±1.1)
	*dnaN-R80A*	41.9 (±1.0) ^b^
	*dnaN-R197A*	45.2 (±1.4) ^b^
	*dnaN-K198E*	49.5 (±1.8)

^***a***^ Values shown represent the average of four separate determinations ± one standard deviation. Growth rates are slightly slower than typical liquid cultures due to culturing in 96 well microtiter plates.

^***b***^ These values are significantly different from the wild type control (*p* < 0.05).

### Mutations targeting loop II and the central pore of the β clamp modestly influence spontaneous mutagenesis

The β clamp interacts with the mismatch repair proteins MutS and MutL [47,48]. Furthermore, based on results of the cryo-electron microscopy (cryo-EM) structure of Pol III core (Pol IIIαεθ) in complex with the C-terminal domain of the τ subunit of DnaX (τ_C_) and the β clamp assembled on DNA, residues 1035–1045 in the OB fold of Pol IIIα interacted with loop I of the β clamp [49]. Importantly, this interaction, among others, was suggested to stabilize the Pol III core-β clamp complex, facilitating processive DNA replication. In order to determine whether loop I, loop II and/or the central pore of the clamp contribute to replication fidelity, through an effect on either Pol III or MutS/MutL function, we measured the frequency of spontaneous mutation for each mutant *dnaN* strain using Rif^R^. Since MMR corrects most replication errors before they become mutations (reviewed in [50,51]), we measured spontaneous mutation frequencies in both *mutL*^*+*^ and Δ*mutL* genetic backgrounds. In the *mutL*^*+*^ background, the frequency for each *dnaN* mutant was comparable, within error, to the wild type control (Table 5), consistent with loop I, loop II and the central pore of the β clamp being dispensable for MMR. However, in the Δ*mutL* background, the frequency of spontaneous Rif^R^ in the *dnaN-Q149A* mutant was ~2-fold higher than the wild type control, while the *dnaN-K12E* and *dnaN-K198E* mutant strains were ~2-fold lower than wild type. Although small, these ~2-fold changes were statistically significant (see 95% CI in Table 5). Spontaneous mutation frequencies for the other Δ*mutL dnaN* mutant strains were indistinguishable from the *dnaN*^*+*^ control. Residues Q149 and K198 interact with DNA, while K12 is immediately adjacent to several residues that contact the DNA ([27]; see Fig 1). These findings suggest that β clamp-DNA interactions contribute modestly to the fidelity of Pol III replication, and argue that the proposed Pol IIIα OB fold-β clamp loop I interaction is either dispensable for high fidelity replication in the absence of DNA damage, or that single alanine amino acids substitutions within loop I fail to perturb the Pol IIIα-β clamp loop I interaction.

**Table 5:** Effects of mutant *dnaN* alleles on spontaneous mutagenesis.

Mutant class	*dnaN* allele	Spontaneous mutation frequency: ^a^	
		*mutL*^*+*^	Δ*mutL*::*cat*
**Control**	*dnaN*^*+*^	6.07 x 10^−8^ (1.67 x 10^−8^–1.23 x 10^−7^)	9.39 x 10^−6^ (8.41 x 10^−6^–1.29 x 10^−5^)
**Loop I**	*dnaN-P20A*	3.31 x 10^−8^ (1.79 x 10^−8^–1.11 x 10^−7^)	1.50 x 10^−5^ (7.69 x 10^−6^–2.31 x 10^−5^)
	*dnaN-L21A*	1.09 x 10^−8^ (8.80 x 10^−9^–5.26 x 10^−8^)	1.54 x 10^−5^ (1.30 x 10^−5^–1.92 x 10^−5^)
	*dnaN-G22A*	5.30 x 10^−8^ (1.52 x 10^−8^–1.20 x 10^−7^)	1.26 x 10^−5^ (6.40 x 10^−6^–2.91 x 10^−5^)
	*dnaN-G23A*	1.51 x 10^−8^ (8.80 x 10^−9^–3.55 x 10^−8^)	1.16 x 10^−5^ (8.45 x 10^−6^–1.76 x 10^−5^)
	*dnaN-R24A*	6.76 x 10^−8^ (2.17 x 10^−8^–1.43 x 10^−7^)	1.08 x 10^−5^ (4.62 x 10^−6^–1.62 x 10^−5^)
	*dnaN-P25A*	5.76 x 10^−8^ (2.03 x 10^−8^–7.35 x 10^−8^)	9.53 x 10^−6^ (7.50 x 10^−6^–1.94 x 10^−5^)
	*dnaN-T26A*	8.62 x 10^−8^ (1.10 x 10^−8^–3.02 x 10^−7^)	1.37 x 10^−5^ (1.10 x 10^−5^–1.70 x 10^−5^)
	*dnaN-L27A*	2.66 x 10^−8^ (1.34 x 10^−8^–1.04 x 10^−7^)	8.49 x 10^−6^ (4.94 x 10^−6^–1.14 x 10^−5^)
**Loop II**	*dnaN-H148A*	5.51 x 10^−8^ (1.33 x 10^−8^–1.48 x 10^−7^)	2.35 x 10^−5^ (1.20 x 10^−5^–5.60 x 10^−5^)
	*dnaN-Q149A*	4.00 x 10^−8^ (1.49 x 10^−8^–1.35 x 10^−7^)	1.95 x 10^−5^ (1.50 x 10^−5^–2.83 x 10^−5^) ^b^
	*dnaN-V151A*	2.72 x 10^−8^ (2.08 x 10^−8^–5.63 x 10^−8^)	9.75 x 10^−6^ (7.63 x 10^−6^–2.54 x 10^−5^)
	*dnaN-Y153A*	1.81 x 10^−8^ (1.09 x 10^−8^–7.35 x 10^−8^)	2.05 x 10^−5^ (1.24 x 10^−5^–3.23 x 10^−5^)
	*dnaN-Y154A*	2.02 x 10^−8^ (1.16 x 10^−8^–1.17 x 10^−7^)	2.57 x 10^−5^ (1.18 x 10^−5^–3.95 x 10^−5^)
**Central pore**	*dnaN-K12E*	5.95 x 10^−8^ (5.00 x 10^−8^–1.52 x 10^−7^)	4.60 x 10^−6^ (2.78 x 10^−6^–5.21 x 10^−6^) ^b^
	*dnaN-Q15A*	1.46 x 10^−7^ (3.29 x 10^−8^–3.36 x 10^−7^)	1.99 x 10^−5^ (1.09 x 10^−5^–4.18 x 10^−5^)
	*dnaN-Q16A*	2.94 x 10^−8^ (1.29 x 10^−8^–1.43 x 10^−7^)	8.12 x 10^−6^ (4.55 x 10^−6^–1.30 x 10^−5^)
	*dnaN-R73A*	1.10 x 10^−7^ (4.31 x 10^−8^–2.60 x 10^−7^)	6.58 x 10^−6^ (3.29 x 10^−6^–8.55 x 10^−6^)
	*dnaN-R80A*	3.21 x 10^−8^ (1.45 x 10^−8^–9.19 x 10^−8^)	8.88 x 10^−6^ (5.07 x 10^−6^–1.74 x 10^−5^)
	*dnaN-R197A*	7.81 x 10^−8^ (2.66 x 10^−8^–1.39 x10^-7^)	4.83 x 10^−6^ (2.15 x 10^−6^–1.35 x 10^−5^)
	*dnaN-K198E*	1.03 x 10^−7^ (3.07 x 10^−8^–1.46 x 10^−7^)	5.45 x 10^−6^ (3.33 x 10^−6^–7.58 x 10^−6^) ^b^

^***a***^ The frequency of spontaneous mutation was measured as described [46]. Values represent the median of 9 independent determinations. 95% confidence intervals (CI) are indicated [41].

^***b***^ These values are significantly different from the wild type control based on 95% CI.

### Distinct roles for loop I, loop II and the central pore of the β clamp in coping with DNA damage

**Fig 3:**
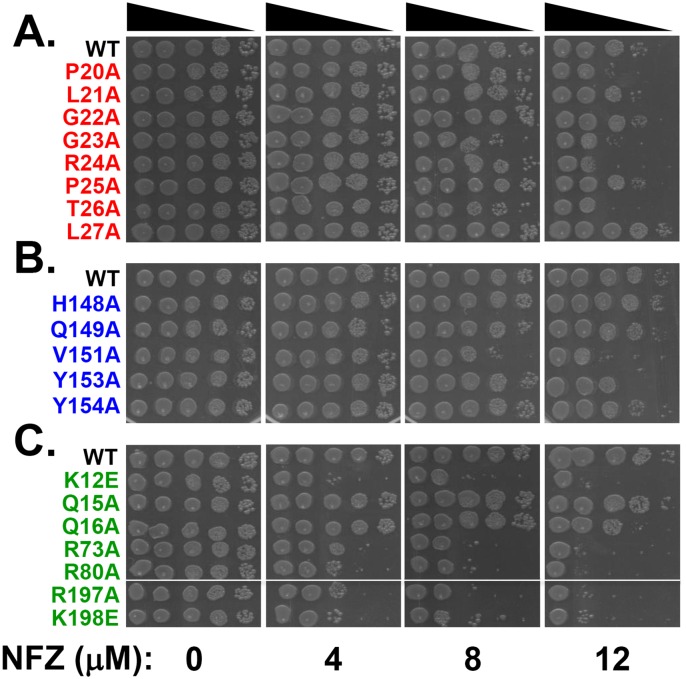
Sensitivity of mutant *dnaN* strains to NFZ. NFZ sensitivity of *dnaN* strains bearing mutations in **(A)** loop I (red), **(B)** loop II (blue) or **(C)** the central pore of the β clamp (green) was measured as described in *Materials and Methods*. Dimethylformamide (DMF) was used for the 0 NFZ control. This experiment was performed 4 times with 2 separate clones. Representative results shown.

To gain insight into whether β clamp-DNA interactions contribute to DNA repair and/or DNA damage tolerance, we measured the sensitivity of each *dnaN* mutant to nitrofurazone (NFZ), methyl methanesulfonate (MMS), ultraviolet light (UV) and hydrogen peroxide (H_2_O_2_). We chose these agents since repair of the DNA damages that they collectively induce requires the majority of the known DNA repair and damage tolerance functions, including TLS by Pol II, Pol IV and Pol V (reviewed in [50]). To measure sensitivity to NFZ and MMS, we spotted 10 μl aliquots of 10-fold serial dilutions of exponential cultures onto agar plates supplemented with the indicated concentrations of the respective chemical. We began with NFZ. As summarized in Fig 3, more than half of the mutants were sensitized to NFZ, including *dnaN-P20A*, *dnaN-G23A*, *dnaN-R24A* and *dnaN-T26A* in loop I, *dnaN-V151A* and *dnaN-Y153A* in loop II, and *dnaN-K12E*, *dnaN-Q16A*, *dnaN-R73A*, *dnaN-R80A*, *dnaN-R197A* and *dnaN-K198E* lining the central pore. Of these 12, 5 corresponded to positions in the β clamp known to contact DNA, including R24, Y153, R73, R80 and K198 [27]. Furthermore, mutations mapping to loop II (V151A), and in particular the central pore (K12E, R73A, R80A, R197A & K198E), displayed a far greater level of NFZ sensitivity than mutations mapping to other positions in the β clamp, suggesting these regions play a more prominent role in coping with NFZ-induced DNA damage.

**Fig 4:**
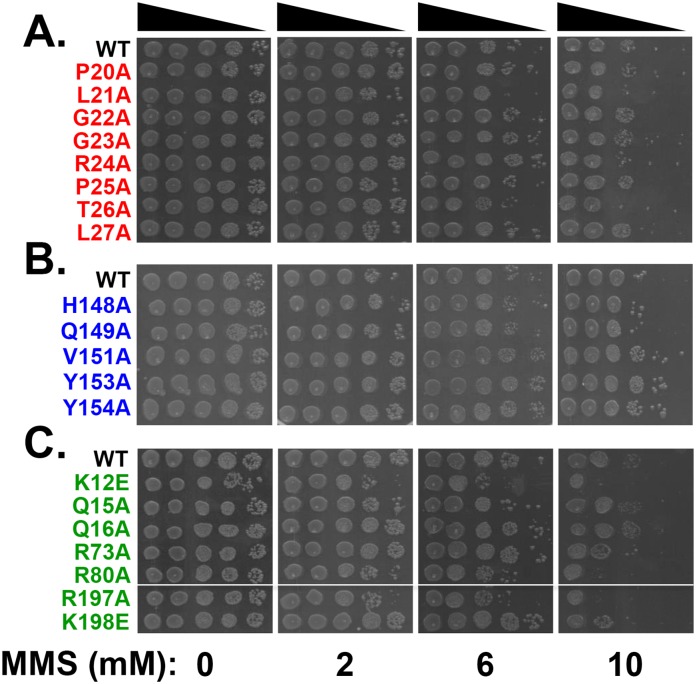
Sensitivity of mutant *dnaN* strains to MMS. MMS sensitivity of *dnaN* strains bearing mutations in **(A)** loop I (red), **(B)** loop II (blue) or **(C)** the central pore of the β clamp (green) was measured as described in *Materials and Methods*. This experiment was performed 4 times with 2 separate clones. Representative results shown.

Compared to NFZ, fewer mutants were sensitized to MMS, including *dnaN-L21A* and *dnaN-T26A* in loop I and *dnaN-K12E*, *dnaN-R80A* and *dnaN-R197A* lining the central pore (Fig 4). Of these, only R80 is known to contact DNA [27]. Interestingly, none of the mutations mapping to loop II conferred sensitivity to MMS. It is noteworthy that with the exception of *dnaN-L21A*, those sensitized to MMS were also sensitized to NFZ (summarized in Table 1). Interestingly, *dnaN-V151A*, *dnaN-Y153A* and *dnaN-Y154A* appeared modestly resistant to MMS compared to the wild type control (Fig 4), suggesting these mutant clamps are able to cope more efficiently with MMS-induced DNA damage than wild type β.

To measure UV sensitivity, we spotted 10 μl aliquots of 10-fold serial dilutions of exponential cultures onto agar plates, allowed them to dry then irradiated with the indicated dose of UV. As summarized in S2 Fig, none of the *dnaN* mutants were sensitized to UV. Likewise, based on results of a disk diffusion assay [39], none of the *dnaN* mutants conferred sensitivity to H_2_O_2_ (S3 Fig). In summary, a total of 13 *dnaN* mutants displayed sensitivity to NFZ and/or MMS, with none conferring sensitivity to UV or H_2_O_2_.

### Loop I, loop II and the central pore of the β clamp make distinct contributions to DNA damage-induced mutagenesis

**Fig 5:**
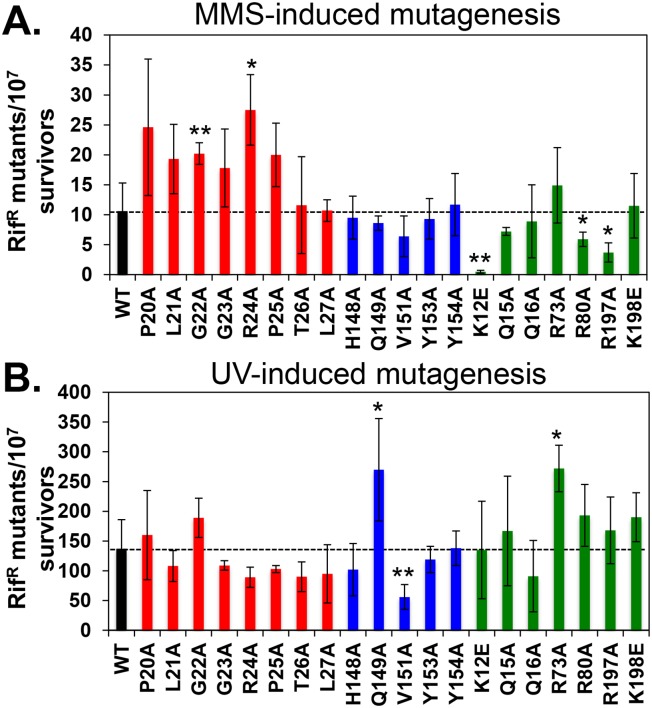
Ability of mutant *dnaN* strains to support DNA damage-induced mutagenesis. Frequencies of **(A)** MMS-induced (2 mM) and **(B)** UV-induced mutagenesis (60 J/m^2^) were measured as described in *Materials and Methods*. Results represent the average of 3 independent determinations using 2 independent clones for each *dnaN* allele ± one standard deviation. Bars are color coded to indicate mutations analyzed map to loop I (red), loop II (blue) or the central pore (green) of the β clamp. Symbols: *, *p* < 0.05; **, *p* < 0.001.

We next asked whether any of the *dnaN* mutants were impaired for supporting DNA damage-induced mutagenesis. The NFZ and MMS sensitivity observed for several of the *dnaN* mutants discussed above suggested a possible Pol IV defect. Most DNA damage-induced mutagenesis in *E*. *coli* is dependent on Pol V, including that induced by MMS and UV ([52,53]; reviewed in [50]). Although Pol IV is inefficient at catalyzing bypass of UV-induced DNA lesions [54], it plays a biologically important role in accurate bypass of MMS-induced lesions, including *N*^*3*^-methyladenine (*N*^*3*^-mdA) [37,55–57]. Thus, the frequency of MMS-induced mutagenesis is a reflection of the relative activities of Pol IV and Pol V. For example, the frequency of MMS-induced mutagenesis is increased by more than 10-fold in the absence of Pol IV function [37,55]. Since we hypothesized that the NFZ and MMS sensitive *dnaN* mutants were potentially impaired for Pol IV function, we measured frequencies of mutagenesis induced by MMS or UV. We first measured frequencies for MMS-induced mutagenesis. A total of 5 *dnaN* mutants displayed an altered frequency of MMS-induced mutagenesis, including *dnaN-G22A* and *dnaN-R24A* mapping to loop I, and *dnaN-K12E*, *dnaN-R80A* and *dnaN-R197A* mapping to the central pore (Fig 5A). Of these 5, only R24 and R80 are known to interact with DNA [27]. Interestingly, none of the mutations in loop II affected the frequency of MMS-induced mutagenesis. The mutations in loop I (G22A & R24A) displayed a ~2- to ~3-fold increase in their respective frequencies of MMS-induced mutagenesis, while the mutations in the central pore were impaired either completely (K12E), or by ~50% relative to the wild type control (R80A & R197A) (Fig 5A). While neither loop I mutant was sensitized to MMS, all 3 central pore mutants were (Fig 4), which may contribute to their reduced frequency of MMS-induced mutagenesis. To determine whether MMS sensitivity of the *dnaN-K12E* mutant influenced its ability to support induced mutagenesis, we measured mutation frequency over a range of MMS concentrations (0.5 to 2.0 mM). As summarized in S4 Fig, the *dnaN-K12E* mutant failed to display induced mutagenesis at all MMS concentrations examined. Finally, as discussed below, the 3 *dnaN* mutants that were impaired for MMS-induced mutagenesis (*dnaN-K12E*, *dnaN-R80A* & *danN-R197A*) were proficient for UV-induced mutagenesis (Fig 5B), indicating that their MMS defect was not the result of a failure to induce SOS, or to properly manage the actions of Pol V.

We next measured respective frequencies of UV-induced mutagenesis. In contrast to its role following exposure to MMS, mutations targeting loop I failed to significantly affect the frequency of UV-induced mutagenesis. Instead, 2 mutations in loop II (*dnaN-Q149A* & *dnaN-V151A*) and one in the central pore (*dnaN-R73A*) displayed altered frequencies (Fig 5B). The *dnaN-Q149A* and *dnaN-R73A* mutants exhibited a ~2-fold higher UV-induced mutation frequency compared to the wild type control, while the frequency of the *dnaN-V151A* mutant was reduced by ~50% (Fig 5B). Importantly, residues Q149 and R73 are known to interact with the DNA template [27]. Taken together, findings discussed above indicate that substitutions in loop I, loop II and the central pore exert modest, albeit distinct effects on MMS- and UV-induced mutagenesis. Several of these mutations map to β clamp residues that make direct contact with the DNA template, providing support for the view that β clamp-DNA interactions contribute to the efficiency of TLS.

### The modestly increased frequency of MMS-induced mutagenesis of the *dnaN-G22A* and *dnaN-R24A* mutants is dependent on *dinB* (Pol IV) function

**Fig 6:**
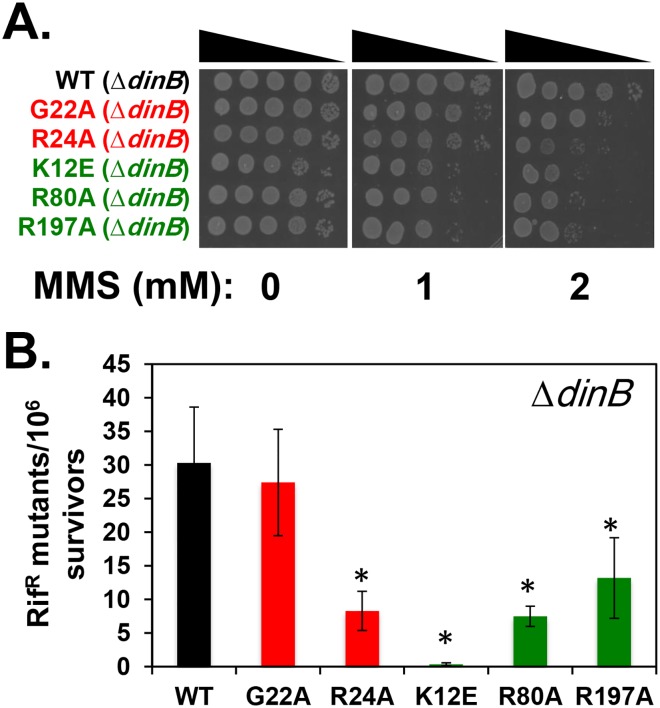
Epistasis of *dnaN-G22A* and *dnaN-R24A* with Δ*dinB*. Double mutants bearing Δ*dinB* and the indicated *dnaN* allele were examined for **(A)** MMS sensitivity and **(B)** MMS-induced mutagenesis (1.0 mM) as described in *Materials and Methods*. Results presented in panel B represent the average of 3 determinations from two independent clones for each strain ± one standard deviation. Symbols: *, *p* < 0.05.

Since Pol IV function attenuates the magnitude of Pol V-dependent mutagenesis in response to MMS, we hypothesized that the increased frequency of MMS-induced mutagenesis observed for the *dnaN-G22A* and *dnaN-R24A* mutants (Fig 5A) was the result of a partially impaired ability of these 2 mutant β clamps to properly manage the actions of Pol IV, leading to more robust levels of Pol V dependent mutagenesis. We likewise questioned whether the reduced frequencies of mutagenesis observed for the *dnaN-K12E*, *dnaN-R80A* and/or *dnaN-R197A* mutant strains (Fig 5B) were related to altered Pol IV function. As a test of these hypotheses, we engineered double mutant strains expressing the mutant *dnaN* alleles together with Δ*dinB*, and measured their respective abilities to cope with MMS. We first measured MMS sensitivity of the double mutants using the same spot assay discussed above. Although the *dnaN-G22A* and *dnaN-R24A* mutants failed to exhibit MMS sensitivity when assayed in the *dinB*^*+*^ background (Fig 4), they were both sensitized to MMS in the Δ*dinB* background (Fig 6A). These results suggest that Pol IV contributes to the ability of these mutants to cope with MMS-induced DNA damage. In contrast to *dnaN-G22A* and *dnaN-R24A*, the *dnaN-K12E*, *dnaN-R80A* and *dnaN-R197A* mutants exhibited similar levels of MMS sensitivity compared to the wild type control, irrespective of *dinB* function (compare Figs 4 & 6A), indicating their MMS sensitivity was independent of Pol IV.

We next measured MMS-induced mutagenesis. Whereas the frequency of MMS-induced mutagenesis of the *dnaN-G22A* mutant was indistinguishable from the wild type control, that of *dnaN-R24A* was reduced to ~30% of the control (Fig 6B). Thus, the increased frequency of MMS-induced mutagenesis observed for the *dnaN-G22A* and *dnaN-R24A* mutants was completely dependent on Pol IV function. In contrast to the loop I mutants, which were to varying degrees epistatic with Δ*dinB*, the inability of the *dnaN-K12E*, *dnaN-R80A* and *dnaN-R197A* mutants to support efficient MMS-induced mutagenesis was unrelated to *dinB* function (compare Figs 5A & 6B). Taken together, these results demonstrate a role for positions G22 and R24 in loop I of the β clamp in ensuring the efficiency of Pol IV TLS *in vivo*. In contrast, the impaired ability of the central pore mutants bearing substitution of positions K12, R80 and R197 to support MMS-induced mutagenesis is likely the result of their MMS hypersensitivity.

### Loop II and the central pore of the β clamp, and possibly loop I contribute to *hda* function

**Fig 7:**
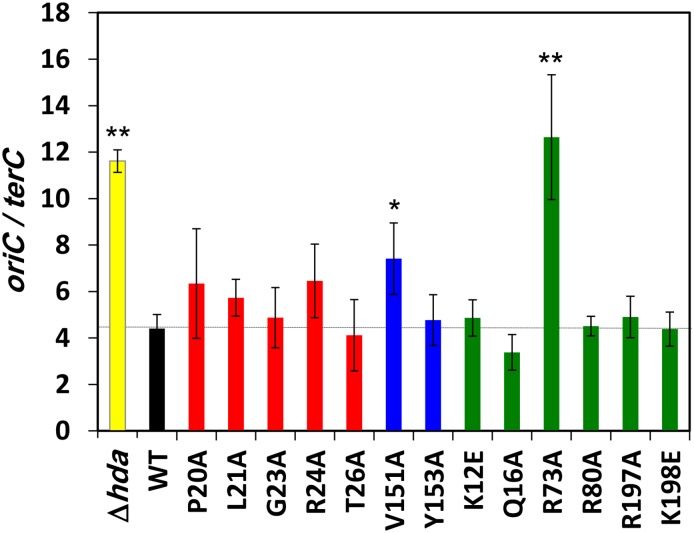
*oriC*/*terC* ratios in the different *dnaN* mutant strains. The *oriC*/*terC* ratio in the indicated mutant *dnaN* strains was measured as described in *Materials and Methods*. Results represent the average of 3 determinations ± one standard deviation. Symbols: *, *p* < 0.05; **, *p* < 0.001.

Several of the *dnaN* mutants that exhibited sensitivity to NFZ and/or MMS were proficient for DNA-damage induced mutagenesis, indicating that they were impaired for one or more functions distinct from TLS. Loss of *seqA* function leads to UV sensitivity due to over initiation of DNA replication [58]. Since Hda interacts with β camp to regulate activity of DnaA in initiation [34], we hypothesized that one or more of the *dnaN* mutants that were sensitized to NFZ and/or MMS may be impaired for *hda* function. As a test of this hypothesis, we used qPCR to measure *oriC*/*terC* ratios in these strains. If these *dnaN* mutants were impaired for supporting *hda* function then we would expect them to posses an elevated *oriC* copy number relative to *terC* due to over initiation. As summarized in Fig 7, loss of *hda* function resulted in an almost 3-fold increase in the *oriC*/*terC* ratio, as expected [34,40]. Likewise, the *oriC*/*terC* ratio was also elevated ~2-fold and ~3-fold, respectively, in the *dnaN-V151A* and *dnaN-R73A* strains. Although failing to reach statistical significance, *oriC*/*terC* ratios were also modestly elevated in the *dnaN-P20A*, *dnaN-L21A* and *dnaN-R24A* strains. Residues R24 and R73 interact with DNA, while P20, L21 and V151 failed to do so in the crystal [27]. Taken together, these findings suggest that *dnaN-V151A* and *dnaN-R73A* are impaired for regulating initiation, consistent with a role for β clamp-DNA interactions in supporting *hda* function.

## Discussion

**Fig 8:**
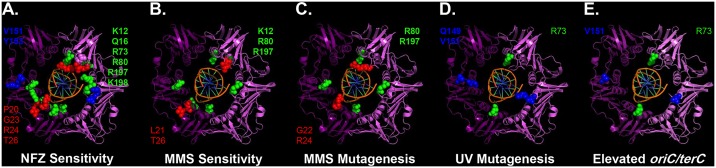
Summary of the major phenotypes observed for *dnaN* mutations. Residues that played a significant role in protecting *E*. *coli* against killing by **(A)** NFZ or **(B)** MMS, or that when substituted significantly affected **(C)** the frequency of mutagenesis induced by exposure to MMS, **(D)** the frequency of mutagenesis induced by exposure to UV light, or **(E)** the ratio of *oriC* to *terC*, suggesting an *hda* defect, are represented on the structure of the β clamp-DNA complex. Residues are color coded by position, with those in loop I colored red, loop II colored blue, and the central pore colored green. This image was generated using the MacPyMol Molecular Graphics System, Ver. 1.7.4 Schrodinger, LLC and the coordinates for the crystal structure of the β clamp-DNA complex (PBD: 3BEP).

The goal of this work was to determine whether residues in the β sliding clamp that directly contact the DNA template contribute to *E*. *coli* viability or its ability to effectively cope with DNA damage by TLS. We analyzed a total of 22 different *dnaN* mutations collectively targeting loop I, loop II and the central pore of the β clamp. Ten of these 22 mutations targeted positions in the β clamp previously demonstrated to contact the DNA template, while the other 12 affected nearby residues [27]. Seventeen of these 22 mutations displayed one or more mutant phenotypes: 2 were unable to support bacterial growth, 3 more were modestly impaired in this regard, 12 were sensitized to NFZ, 5 were sensitized to MMS, 8 displayed modestly altered frequencies of DNA damage-induced mutagenesis and 2 appear to be impaired for supporting *hda* function (summarized in Table 1 & Fig 8). With the notable exceptions of D150 and R152, both of which failed to support *E*. *coli* viability, the other *dnaN* mutations supported normal growth, arguing they retained the ability to be loaded onto DNA *in vivo* well enough to support chromosomal replication. Taken together, these results demonstrate that discrete β clamp-DNA interaction regions contribute to the ability of *E*. *coli* to tolerate DNA damage (Fig 8A & 8B). While the individual β clamp-DNA interactions were largely dispensable for DNA damage-induced mutagenesis, they nevertheless contribute significantly to its efficiency *in vivo* (Fig 8C & 8D). Finally, we acknowledge the possibility that β clamp-DNA interactions contribute more significantly to TLS than suggested here by phenotypes of single amino acid substitutions in the clamp.

Loop II of the β clamp was unique among the 3 surfaces examined in this work in that it was the only region in which mutations (D150A & R152A) failed to support *E*. *coli* viability (Table 2). Although viable, the *dnaN-G23A*, *dnaN-R24A* mutants in loop I, and the *dnaN-V151A* mutant in loop II were each constructed ~10-fold less efficiently compared to the wild type control (Table 2). We suggest this is due to each of these mutants being impaired for one or more β clamp functions that become essential for viability under specific circumstances not routinely encountered during growth. In contrast to β-R24A, which was modestly impaired for loading onto DNA *in vitro*, the ability of DnaX to load the mutant β-R152A clamp protein was not examined. Thus, it is unclear whether the inability of the *dnaN-R152A* mutant to support viability is related to a loading defect. Regardless, neither D150 nor V151 of the β clamp interacted with DNA in the β clamp-DNA crystal [27]. Neuwald suggested that positions Q149 and K198 sense DNA within the central pore of the β clamp and relay this signal to residues D150 and R152 as part of the clamp loading mechanism [59]. Thus, the inability of *dnaN-D150A* and *dnaN-R152A* to support *E*. *coli* viability may relate to a loading defect. Alternatively, it is possible that these residues perform one or more roles critical to *E*. *coli* viability in addition to loading the β clamp onto DNA.

Whereas Pol IV catalyzes accurate bypass of MMS-induced DNA lesions, Pol V is responsible for most mutations resulting from MMS exposure [52,53,55]. We observed a small yet statistically significant ~2- to ~3-fold increase in the frequency of MMS-induced mutagenesis for the *dnaN-G22A* and *dnaN-R24A* mutants compared to the wild type *dnaN* control (Fig 5A). Importantly, this increase was absolutely dependent on *dinB* (Pol IV) function (Fig 6B). Taken together, these findings suggest that the G22A and R24A substitutions either impair the efficiency with which Pol IV is recruited to sites of MMS-induced DNA damage, or interfere with the ability of the β clamp to properly manage the actions of Pol IV after it is recruited. As a result, Pol V plays a greater role in bypassing MMS-induced lesions, explaining the ~2- to ~3-fold increase in mutation frequency (Fig 5A). Although G22 of the β clamp failed to contact DNA in the crystal [27], flexibility at this position may contribute to the ability of R24 to interact with DNA [60–62]. In this case, the modest Pol IV defect observed for *dnaN-G22A* and *dnaN-R24A* could result from impaired β clamp-DNA interactions. Alternatively, Pol IV may contact loop I of the β clamp, and the G22A and R24A mutations may interfere with this interaction. Regardless, it is important to point out that the *dnaN-G22A* and *dnaN-R24A* mutants retained a partial ability to manage Pol IV, since their respective frequencies of MMS-induced mutagenesis were still higher than the isogenic Δ*dinB* strain (~20 x 10^−7^ and ~27 x 10^−7^ for the *dnaN* mutants in the presence of *dinB*^*+*^, respectively, compared to ~30 x 10^−6^ for the *dnaN*^*+*^ Δ*dinB* strain; see Figs 5 & 6). Finally, in addition to their role in Pol IV function, positions G22 and R24 in loop I of the β clamp appear to play an additional role in protecting *E*. *coli* against killing by MMS independent of Pol IV function (Fig 6A).

We previously presented evidence that β^148–152^ (bearing alanine substitutions of residues H148-R152) was impaired for Pol V mutagenesis *in vivo* [28,30]. Consistent with this view, two of the mutations analyzed in this work that mapped to loop II of the β clamp significantly altered the frequency of UV-induced mutagenesis (Fig 5B). The mutation frequency of *dnaN-Q149A* was elevated ~2-fold compared to the wild type control, while that of *dnaN-V151A* was reduced ~2-fold. Patoli *et al*. determined that position N359 of the UmuC subunit of Pol V hydrogen bonds to residue R152 of the β clamp [63]. R152 of the symmetry-related β molecule interacted with DNA [27]. Thus, Pol V and the DNA may compete with each other for binding loop II of the β clamp. The ~2-fold increase in UV-induced mutagenesis observed for the *dnaN-Q149A* mutant may result from a DNA binding defect, shifting the equilibrium away from β clamp-DNA interaction and towards β clamp-Pol V interaction. Likewise, the ~2-fold reduction in mutation frequency for the *dnaN-V151A* mutant may reflect the reciprocal situation in which the mutation impairs the β clamp-Pol V interaction. Alternatively, since the β clamp functions as a homodimer, R152 in one β protomer may interact with UmuC, while R152 in the other β protomer may contact the DNA template. In this case, altered Pol V function *in vivo* may result from effects on β clamp-DNA interactions, as well as secondary effects of these mutations on β clamp-Pol V interactions. Finally, β^148-152^ was impaired for interaction with both Pol II and Pol IV *in vitro*, and was unable to support processive replication by these Pols *in vitro* [28]. Although the single substitution mutations used here failed to exert an obvious effect on Pol IV function *in vivo*, our prior *in vivo* and *in vitro* results with β^148-152^ [28], taken together with our *in vivo* findings presented here, indicate that loop II of the β clamp contributes to function of all 3 *E*. *coli* TLS Pols. Taken together, these findings suggest that multiple substitutions within loop II may be required to observe a Pol II and Pol IV defect *in vivo*. These findings additionally suggest that there are significant differences in how these 3 TLS Pols interact with loop II of the β clamp.

While mutations mapping to loop I and loop II conferred sensitivity to NFZ, sensitivity of the *dnaN* mutations mapping to the central pore was particularly robust (Fig 3). NFZ-induced lesions are either repaired by nucleotide excision repair (NER) or bypassed accurately by Pol IV [38,64,65]. All 6 of the NFZ sensitive *dnaN* mutations mapping to the central pore were proficient for NER, based on their ability to tolerate UV light (S2 Fig). Likewise, 3 (Q16A, R73A & K198A) of the 6 were tolerant of MMS, indicating they were largely proficient for Pol IV function. Thus, the mechanistic basis for the NFZ sensitivity of these *dnaN* mutations is unclear. Ona *et al*. demonstrated that exposure of *E*. *coli* to high concentrations of NFZ (200 μM) for short periods of time inhibited DNA replication without causing DNA damage [64]. Thus, the *dnaN* mutants that were sensitized to NFZ may be impaired for interaction with one or more subunits of Pol III, resulting in a less stable replisome that fails to cope efficiently with NFZ. The other 3 *dnaN* mutations (K12E, R80A & R197A) conferring NFZ sensitivity were significantly sensitized to MMS, and were impaired to varying degrees for MMS-induced mutagenesis (Fig 5A). That these 3 *dnaN* mutants were proficient in UV-induced mutagenesis (Fig 5B) argues that their inability to support MMS-induced mutagenesis was the result of their MMS hypersensitivity. The mechanistic basis for this MMS sensitivity is currently unknown, but could be attributable to a Pol III and/or Pol I replication defect. Finally, it is possible that the *dnaN* mutants with increased sensitivity to NFZ and/or MMS are less stable on DNA, or are more rapidly unloaded, possibly due to their impaired ability to interact with one or more partner proteins. This would lead to fewer clamps on DNA, which could reduce the efficiency with which NFZ- and MMS-induced lesions are repaired. Consistent with this model, all 7 of the *dnaN* mutants that grew more quickly than the wild type strain were sensitive to NFZ, and several were sensitive to MMS. Faster recycling of these mutant β clamp proteins off of the DNA and into the soluble pool used by DnaX to repeatedly load clamp onto lagging strand could also lead to more rapid replication, explaining their increased growth rate (Table 1). Furthermore, if these mutant clamps did in fact support faster replication, then the forks in these mutants might collide more frequently with either unrepaired lesions or ssDNA gaps generated during the repair process. This, in turn, would contribute to increased sensitivity to these DNA damaging agents.

While increased sensitivity to DNA damage is typically thought to be the result of an impaired ability to properly cope with DNA damage using accurate repair or potentially mutagenic TLS, it can also be the result of over initiation of DNA replication. In this case, sensitivity results from more frequent collisions between the closely spaced replication forks and DNA lesions or repair intermediates [58,66]. In order to determine whether any of the 13 *dnaN* mutants that were sensitized to NFZ and/or MMS were impaired for supporting *hda* function, we used qPCR to measure their *oriC*/*terC* ratios. As summarized in Table 1 and Fig 7E, *dnaN-V151A* and *dnaN-R73A* exhibited a significantly increased *oriC* copy number compared to the *dnaN*^*+*^ control. Although not statistically significant, *oriC*/*terC* ratios were also modestly elevated in the *dnaN-P20A*, *dnaN-L21A* and *dnaN-R24A* strains compared to the wild type control, suggesting that loop I of the β clamp play a role in managing the actions of Hda *in vivo*. While the elevated *oriC* copy number observed in these strains suggests that these *dnaN* mutants are impaired for managing the ability of Hda to regulate the activity of DnaA in initiation of DNA replication, we cannot rule out the possibility that this phenotype is due at least in part to these *dnaN* mutants being impaired for supporting Pol III replication. In this case, fewer forks would reach *terC*, contributing to an elevated *oriC*/*terC* ratio.

Nine of the 12 residues that did not interact with DNA in the β clamp-DNA crystal examined in this work contributed to the ability of *E*. *coli* to effectively cope with NFZ- and/or MMS-induced DNA damage (summarized in Table 1 & Fig 8A & 8B). The simplest explanation for these phenotypes is that these residues either contact the DNA, despite the fact that they failed to do so in the crystal, or that their substitution affects the structure of adjacent residues in the β clamp that do contact the DNA [27]. However, this argument fails to explain the MMS phenotypes of the loop I *dnaN-L21A* and *dnaN-T26A* mutants. Residue R24 is the only position in loop I known to contact DNA, yet *dnaN-R24A* failed to exhibit MMS sensitivity. In contrast, *dnaN-L21A* and *dnaN-T26A* each conferred MMS sensitivity (summarized in Table 1 & Fig 8B), arguing it was independent of β clamp-DNA interactions. The *dnaN-P20A*, *dnaN-L21A* and *dnaN-R24A* mutants were slightly elevated for *oriC* relative to *terC* (summarized in Table 1 & Fig 8E). Although these differences relative to the wild type control were not statistically significant, they nevertheless suggest that loop I of the β clamp may contribute to *hda* function, possibly via a physical interaction.

In conclusion, 17 of 22 *dnaN* mutations examined in this work displayed one or more mutant phenotype. While it is possible that some of the mutations may affect interaction of the β clamp with one or more of its partner proteins, these findings nevertheless support a model in which β clamp-DNA interactions contribute to the ability of the clamp to manage the actions of its different partner proteins, particularly Pol IV, Pol V and Hda.

## Supporting Information

S1 Fig Molecular structure of the Δ*dnaN*::(*kan*, *sacB*) allele. Cartoon depiction of the wild type *dnaA-dnaN-recF* operon and Δ*dnaN*::(*kan*, *sacB*) allele. Nucleotide (kb) and minute (min) positions refer to approximate *E*. *coli* chromosomal coordinates. Approximate positions of homology for oligonucleotide primers used for construction of the Δ*dnaN*::(*kan*, *sacB*) allele and its recombineering onto the bacterial chromosome are indicated.
(DOCX)

S2 Fig Mutant *dnaN* strains fail to increase UV sensitivity. UV sensitivity of strains bearing mutations in **(A)** loop I (red), **(B)** loop II (blue) or **(C)** the central pore of the β clamp (green) was measured as described in *Materials and Methods*. This experiment was performed 4 times with 2 separate clones. Representative results shown.
(DOCX)

S3 Fig Mutant *dnaN* strains fail to increase H_2_O_2_ sensitivity. H_2_O_2_ sensitivity of strains bearing mutations in either loop I (red), loop II (blue) or the central pore of the β clamp (green) was measured as described in *Materials and Methods*. Results represent the average of 4 separate determinations ± one standard deviation. Sensitivity of the mutants was not significantly different than that of the *dnaN*^*+*^ wild type (WT) strain (*p* > 0.05).
(DOCX)

S4 Fig Ability of the *dnaN-K12E* mutant to support MMS-induced mutagenesis. The ability of the wild type (MN100-1 & MN100-2) and *dnaN-K12E* (MN114-1 & MN114-2) strains to support MMS-induced mutagenesis was measured as described in *Materials and Methods*. Results represent the average of 3 independent determinations using 2 separate clones ± one standard deviation. Symbols are as follows: *, *p* < 0.05; **, *p* < 0.001.
(DOCX)

S1 Table Bacterial strains and plasmids DNAs used in this study. (DOCX)

S2 Table Oligonucleotides used in this study. (DOCX)
