# Supplementary material for: Identification of β Clamp-DNA Interaction Regions That Impair the Ability of E. coli to Tolerate Specific Classes of DNA Damage
Source: PLoS One. 2016 Sep 29;11(9):e0163643. doi: 10.1371/journal.pone.0163643 (PMC5042465; doi:10.1371/journal.pone.0163643)
Supplement: S1 Fig — Cartoon depiction of the wild type dnaA-dnaN-recF operon and ΔdnaN::(kan, sacB) allele. Nucleotide (kb) and minute (min) positions refer to approximate E. coli chromosomal coordinates. Approximate positions of homology for oligonucleotide primers used for construction of the ΔdnaN::(kan, sacB) allele and its recombineering onto the bacterial chromosome are indicated. (DOCX) [file pone.0163643.s001.docx]

**
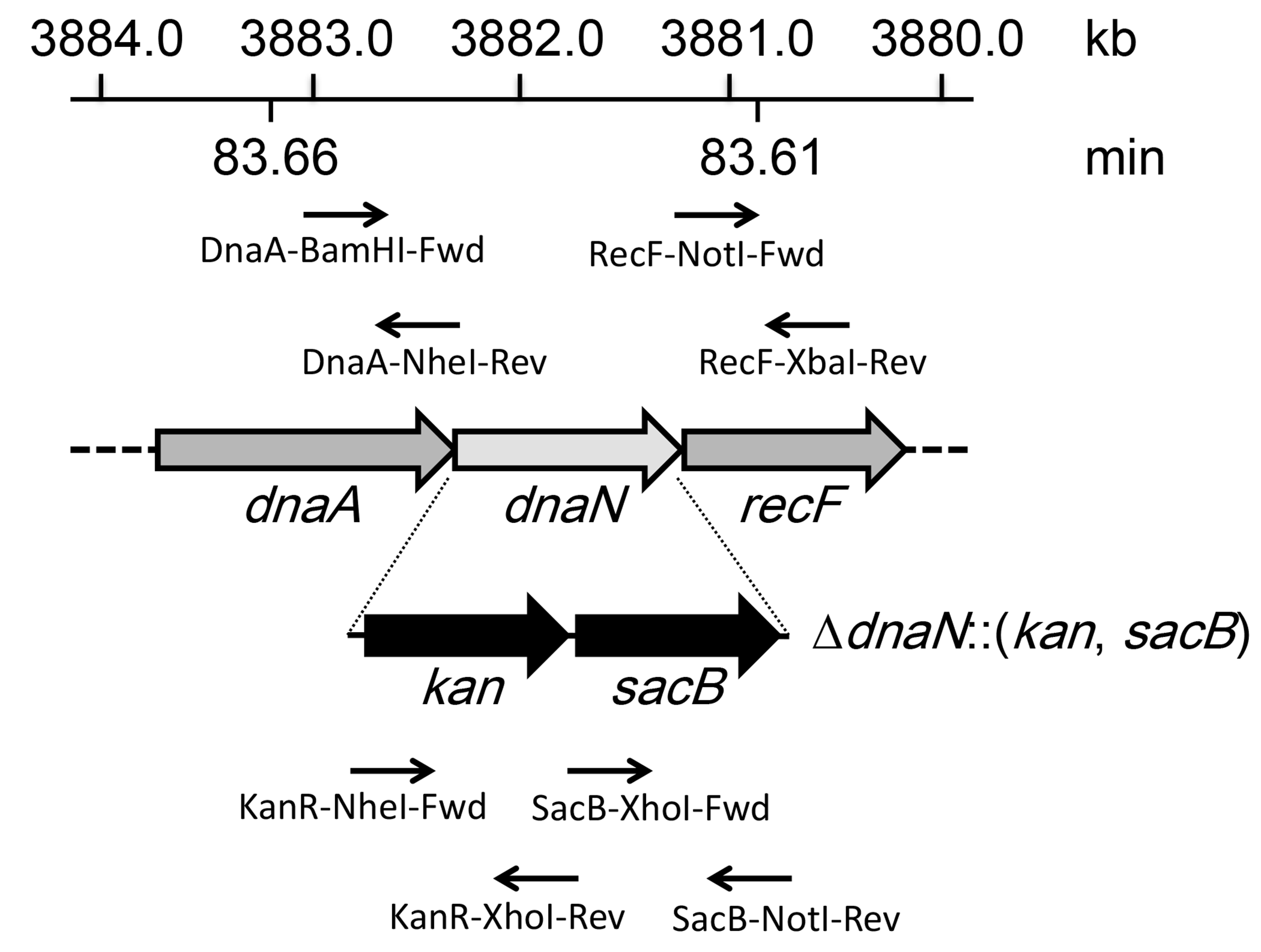
**

**S1 Figure: Molecular structure of the ∆*dnaN*::(*kan*, *sacB*) allele.** Cartoon depiction of the wild type *dnaA-dnaN-recF* operon and ∆*dnaN*::(*kan*, *sacB*) allele. Nucleotide (kb) and minute (min) positions refer to approximate *E. coli* chromosomal coordinates. Approximate positions of homology for oligonucleotide primers used for construction of the ∆*dnaN*::(*kan*, *sacB*) allele and its recombineering onto the bacterial chromosome are indicated.
