# Supplementary material for: Identification of β Clamp-DNA Interaction Regions That Impair the Ability of E. coli to Tolerate Specific Classes of DNA Damage
Source: PLoS One. 2016 Sep 29;11(9):e0163643. doi: 10.1371/journal.pone.0163643 (PMC5042465; doi:10.1371/journal.pone.0163643)
Supplement: S2 Fig — UV sensitivity of strains bearing mutations in (A) loop I (red), (B) loop II (blue) or (C) the central pore of the β clamp (green) was measured as described in Materials and Methods. This experiment was performed 4 times with 2 separate clones. Representative results shown. (DOCX) [file pone.0163643.s002.docx]

**
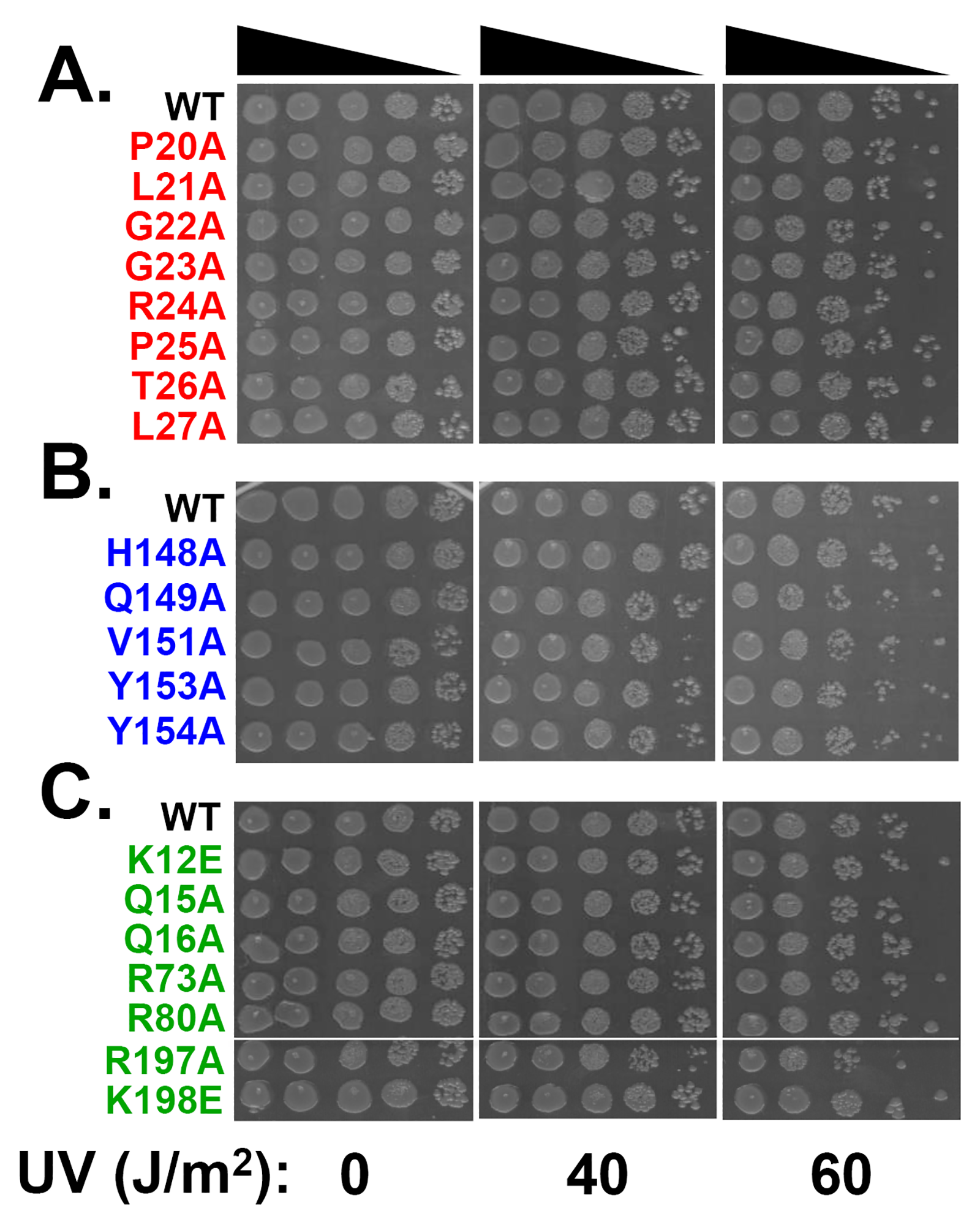
**

**S2 Figure: Mutant *dnaN* strains fail to increase UV sensitivity.** UV sensitivity of strains bearing mutations in **(A)** loop I (red), **(B)** loop II (blue) or **(C)** the central pore of the β clamp (green) was measured as described in *Materials and Methods*. This experiment was performed 4 times with 2 separate clones. Representative results shown.
