# Supplementary material for: Identification of β Clamp-DNA Interaction Regions That Impair the Ability of E. coli to Tolerate Specific Classes of DNA Damage
Source: PLoS One. 2016 Sep 29;11(9):e0163643. doi: 10.1371/journal.pone.0163643 (PMC5042465; doi:10.1371/journal.pone.0163643)
Supplement: S3 Fig — H2O2 sensitivity of strains bearing mutations in either loop I (red), loop II (blue) or the central pore of the β clamp (green) was measured as described in Materials and Methods. Results represent the average of 4 separate determinations ± one standard deviation. Sensitivity of the mutants was not significantly different than that of the dnaN+ wild type (WT) strain (p > 0.05). (DOCX) [file pone.0163643.s003.docx]

**
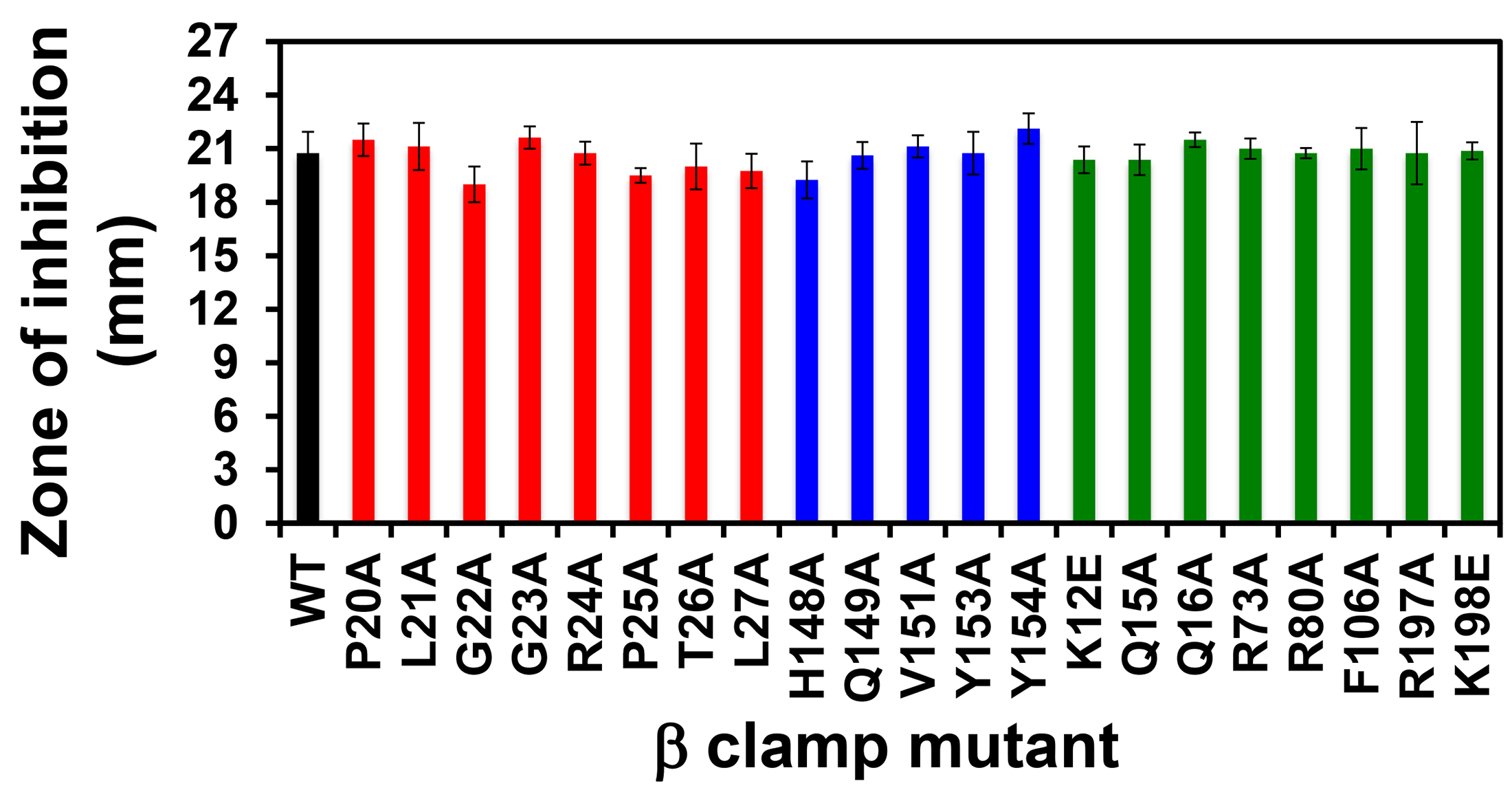
**

**S3 Figure: Mutant *dnaN* strains fail to increase H_2_O_2_ sensitivity.** H_2_O_2_ sensitivity of strains bearing mutations in either loop I (red), loop II (blue) or the central pore of the β clamp (green) was measured as described in *Materials and Methods*. Results represent the average of 4 separate determinations ± one standard deviation. Sensitivity of the mutants was not significantly different than that of the *dnaN^+^* wild type (WT) strain (*p* > 0.05).
