# Supplementary material for: Identification of β Clamp-DNA Interaction Regions That Impair the Ability of E. coli to Tolerate Specific Classes of DNA Damage
Source: PLoS One. 2016 Sep 29;11(9):e0163643. doi: 10.1371/journal.pone.0163643 (PMC5042465; doi:10.1371/journal.pone.0163643)
Supplement: S4 Fig — The ability of the wild type (MN100-1 & MN100-2) and dnaN-K12E (MN114-1 & MN114-2) strains to support MMS-induced mutagenesis was measured as described in Materials and Methods. Results represent the average of 3 independent determinations using 2 separate clones ± one standard deviation. Symbols are as follows: *, p < 0.05; **, p < 0.001. (DOCX) [file pone.0163643.s004.docx]

**
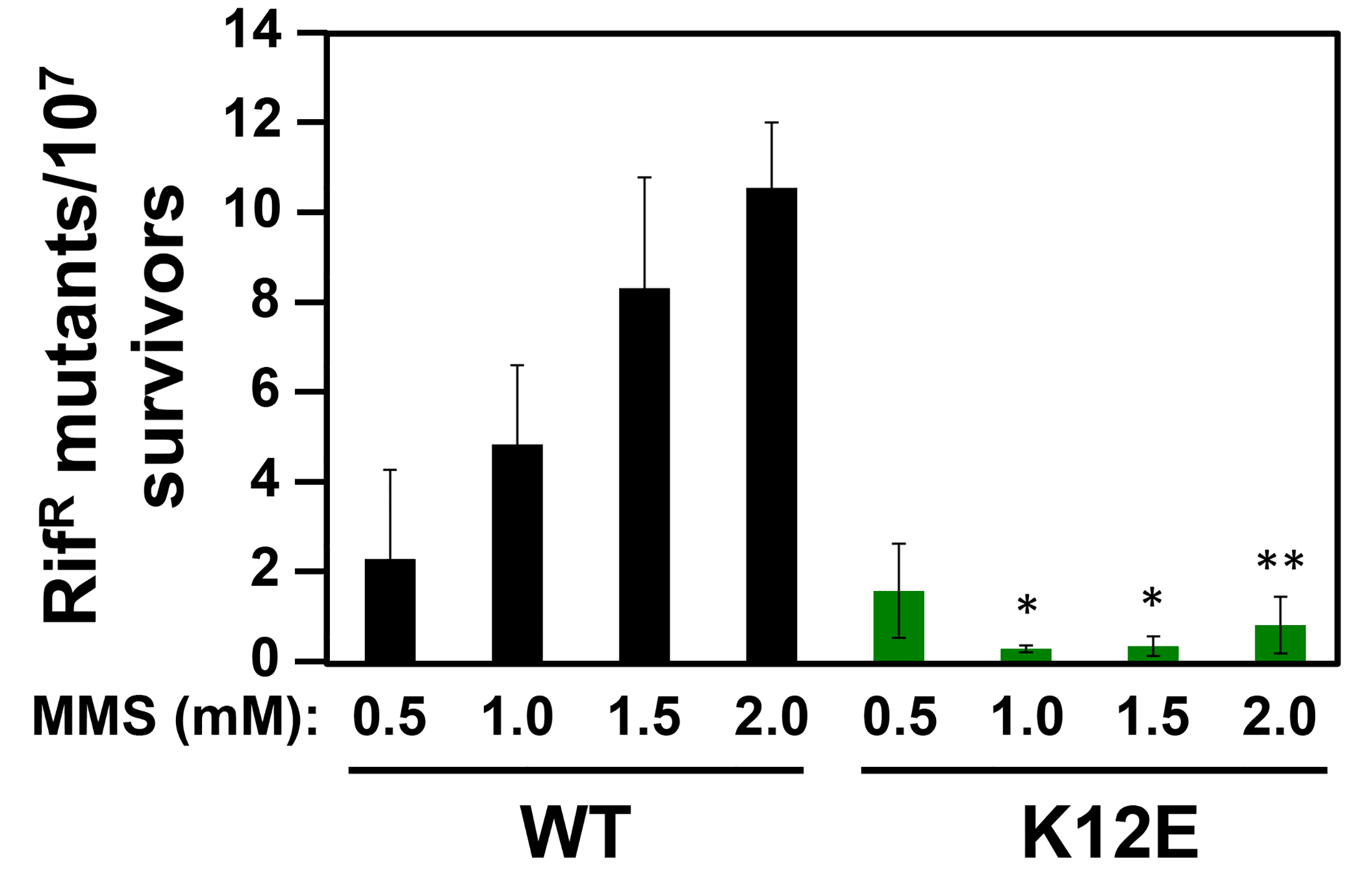
**

**S4 Figure: Ability of the *dnaN-K12E* mutant to support MMS-induced mutagenesis.** The ability of the wild type (MN100-1 & MN100-2) and *dnaN-K12E* (MN114-1 & MN114-2) strains to support MMS-induced mutagenesis was measured as described in *Materials and Methods*. Results represent the average of 3 independent determinations using 2 separate clones ± one standard deviation. Symbols are as follows: *, *p* < 0.05; **, *p* < 0.001.
