# Supplementary material for: Identification of β Clamp-DNA Interaction Regions That Impair the Ability of E. coli to Tolerate Specific Classes of DNA Damage
Source: PLoS One. 2016 Sep 29;11(9):e0163643. doi: 10.1371/journal.pone.0163643 (PMC5042465; doi:10.1371/journal.pone.0163643)
Supplement: S1 Table — (DOCX) [file pone.0163643.s005.docx]

**S1 Table: Bacterial strains and plasmids DNAs used in this study.**

| **Bacterial strains** | | |
| --- | --- | --- |
| **Strain** | **Relevant genotype** | **Source** |
| MG1655 | *ilvG rfb-50 rph-1* | [[1](#_ENREF_1)] |
| MN000 | MG1655: *tnaA300*::Tn*10kan* | Lab stock |
| MS198 | *lamB*::(His_6_-*dnaN^+^*–*cam*) | [[2](#_ENREF_2)] |
| MO90-3 | MG1655: *lamB*::(His_6_-*dnaN^+^*–*cam*) *dnaN^+^* | This work |
| MO90-3-AKSF | MG1655: *lamB*::(His_6_-*dnaN^+^*–*cam*) ∆*dnaN*::(*kan*, *sacB*) | This work |
| MN100-1 | MG1655: ∆*dnaN*::(*kan*, *sacB*) (pJD100) [expresses *dnaN^+^*] | This work |
| MN100-2 | MG1655: ∆*dnaN*::(*kan*, *sacB*) (pJD100) [expresses *dnaN^+^*] | This work |
| MN101-1 | MG1655: ∆*dnaN*::(*kan*, *sacB*) (pMN100) [expresses *dnaN-P20A*] | This work |
| MN101-2 | MG1655: ∆*dnaN*::(*kan*, *sacB*) (pMN100) [expresses *dnaN-P20A*] | This work |
| MN102-1 | MG1655: ∆*dnaN*::(*kan*, *sacB*) (pMN101) [expresses *dnaN-L21A*] | This work |
| MN102-2 | MG1655: ∆*dnaN*::(*kan*, *sacB*) (pMN101) [expresses *dnaN-L21A*] | This work |
| MN103-1 | MG1655: ∆*dnaN*::(*kan*, *sacB*) (pMN102) [expresses *dnaN-G22A*] | This work |
| MN103-2 | MG1655: ∆*dnaN*::(*kan*, *sacB*) (pMN102) [expresses *dnaN-G22A*] | This work |
| MN104-1 | MG1655: ∆*dnaN*::(*kan*, *sacB*) (pMN103) [expresses *dnaN-G23A*] | This work |
| MN104-2 | MG1655: ∆*dnaN*::(*kan*, *sacB*) (pMN103) [expresses *dnaN-G23A*] | This work |
| MN105-1 | MG1655: ∆*dnaN*::(*kan*, *sacB*) (pMN104) [expresses *dnaN-R24A*] | This work |
| MN105-2 | MG1655: ∆*dnaN*::(*kan*, *sacB*) (pMN104) [expresses *dnaN-R24A*] | This work |
| MN106-1 | MG1655: ∆*dnaN*::(*kan*, *sacB*) (pMN105) [expresses *dnaN-P25A*] | This work |
| MN106-2 | MG1655: ∆*dnaN*::(*kan*, *sacB*) (pMN105) [expresses *dnaN-P25A*] | This work |
| MN107-1 | MG1655: ∆*dnaN*::(*kan*, *sacB*) (pMN106) [expresses *dnaN-T26A*] | This work |
| MN107-2 | MG1655: ∆*dnaN*::(*kan*, *sacB*) (pMN106) [expresses *dnaN-T26A*] | This work |
| MN108-1 | MG1655: ∆*dnaN*::(*kan*, *sacB*) (pMN107) [expresses *dnaN-L27A*] | This work |
| MN108-2 | MG1655: ∆*dnaN*::(*kan*, *sacB*) (pMN107) [expresses *dnaN-L27A*] | This work |
| MN109-1 | MG1655: ∆*dnaN*::(*kan*, *sacB*) (pMN108) [expresses *dnaN-H148A*] | This work |
| MN109-2 | MG1655: ∆*dnaN*::(*kan*, *sacB*) (pMN108) [expresses *dnaN-H148A*] | This work |
| MN110-1 | MG1655: ∆*dnaN*::(*kan*, *sacB*) (pMN109) [expresses *dnaN-Q149A*] | This work |
| MN110-2 | MG1655: ∆*dnaN*::(*kan*, *sacB*) (pMN109) [expresses *dnaN-Q149A*] | This work |
| MN111-1 | MG1655: ∆*dnaN*::(*kan*, *sacB*) (pMN111) [expresses *dnaN-V151A*] | This work |
| MN111-2 | MG1655: ∆*dnaN*::(*kan*, *sacB*) (pMN111) [expresses *dnaN-V151A*] | This work |
| MN112-1 | MG1655: ∆*dnaN*::(*kan*, *sacB*) (pMN113) [expresses *dnaN-Y153A*] | This work |
| MN112-2 | MG1655: ∆*dnaN*::(*kan*, *sacB*) (pMN113) [expresses *dnaN-Y153A*] | This work |
| MN113-1 | MG1655: ∆*dnaN*::(*kan*, *sacB*) (pMN114) [expresses *dnaN-Y154A*] | This work |
| MN113-2 | MG1655: ∆*dnaN*::(*kan*, *sacB*) (pMN114) [expresses *dnaN-Y154A*] | This work |
| MN114-1 | MG1655: ∆*dnaN*::(*kan*, *sacB*) (pMN115) [expresses *dnaN-K12E*] | This work |
| MN114-2 | MG1655: ∆*dnaN*::(*kan*, *sacB*) (pMN115) [expresses *dnaN-K12E*] | This work |
| MN115-1 | MG1655: ∆*dnaN*::(*kan*, *sacB*) (pMN116) [expresses *dnaN-Q15A*] | This work |
| MN115-2 | MG1655: ∆*dnaN*::(*kan*, *sacB*) (pMN116) [expresses *dnaN-Q15A*] | This work |
| MN116-1 | MG1655: ∆*dnaN*::(*kan*, *sacB*) (pMN117) [expresses *dnaN-Q16A*] | This work |
| MN116-2 | MG1655: ∆*dnaN*::(*kan*, *sacB*) (pMN117) [expresses *dnaN-Q16A*] | This work |
| MN117-1 | MG1655: ∆*dnaN*::(*kan*, *sacB*) (pMN118) [expresses *dnaN-R73A*] | This work |
| MN117-2 | MG1655: ∆*dnaN*::(*kan*, *sacB*) (pMN118) [expresses *dnaN-R73A*] | This work |
| MN118-1 | MG1655: ∆*dnaN*::(*kan*, *sacB*) (pMN119) [expresses *dnaN-R80A*] | This work |
| MN118-2 | MG1655: ∆*dnaN*::(*kan*, *sacB*) (pMN119) [expresses *dnaN-R80A*] | This work |
| MN119-1 | MG1655: ∆*dnaN*::(*kan*, *sacB*) (pMN120) [expresses *dnaN-R197A*] | This work |
| MN119-2 | MG1655: ∆*dnaN*::(*kan*, *sacB*) (pMN120) [expresses *dnaN-R197A*] | This work |
| MN120-1 | MG1655: ∆*dnaN*::(*kan*, *sacB*) (pMN121) [expresses *dnaN-K198E*] | This work |
| MN120-2 | MG1655: ∆*dnaN*::(*kan*, *sacB*) (pMN121) [expresses *dnaN-K198E*] | This work |
| MKS108 | MG1655: ∆*dinB*::*cam* | This work |
| MN121-1 | MG1655: ∆*dnaN*::(*kan*, *sacB*) ∆*dinB*::*cam* (pJD100) [expresses *dnaN^+^*] | This work |
| MN121-2 | MG1655: ∆*dnaN*::(*kan*, *sacB*) ∆*dinB*::*cam* (pJD100) [expresses *dnaN^+^*] | This work |
| MN122-1 | MG1655: ∆*dnaN*::(*kan*, *sacB*) ∆*dinB*::*cam* (pMN102) [expresses *dnaN-G22A*] | This work |
| MN122-2 | MG1655: ∆*dnaN*::(*kan*, *sacB*) ∆*dinB*::*cam* (pMN102) [expresses *dnaN-G22A*] | This work |
| MN123-1 | MG1655: ∆*dnaN*::(*kan*, *sacB*) ∆*dinB*::*cam* (pMN104) [expresses *dnaN-R24A*] | This work |
| MN123-2 | MG1655: ∆*dnaN*::(*kan*, *sacB*) ∆*dinB*::*cam* (pMN104) [expresses *dnaN-R24A*] | This work |
| MN124-1 | MG1655: ∆*dnaN*::(*kan*, *sacB*) ∆*dinB*::*cam* (pMN115) [expresses *dnaN-K12E*] | This work |
| MN124-2 | MG1655: ∆*dnaN*::(*kan*, *sacB*) ∆*dinB*::*cam* (pMN115) [expresses *dnaN-K12E*] | This work |
| MN125-1 | MG1655: ∆*dnaN*::(*kan*, *sacB*) ∆*dinB*::*cam* (pMN119) [expresses *dnaN-R80A*] | This work |
| MN125-2 | MG1655: ∆*dnaN*::(*kan*, *sacB*) ∆*dinB*::*cam* (pMN119) [expresses *dnaN-R80A*] | This work |
| MN126-1 | MG1655: ∆*dnaN*::(*kan*, *sacB*) ∆*dinB*::*cam* (pMN120) [expresses *dnaN-R197A*] | This work |
| MN126-2 | MG1655: ∆*dnaN*::(*kan*, *sacB*) ∆*dinB*::*cam* (pMN120) [expresses *dnaN-R197A*] | This work |
| RW118 | *lexA^+^* | [[3](#_ENREF_3)] |
| RW542 | *lexA51*(Def) | [[4](#_ENREF_4)] |
| KM52 | ∆*mutL460*::*cam* | [[5](#_ENREF_5)] |
| MN127 | MG1655: ∆*dnaN*::(*kan*, *sacB*) ∆*mutL*::*cam* (pJD100) [expresses *dnaN^+^*] | This work |
| MN128 | MG1655: ∆*dnaN*::(*kan*, *sacB*) ∆*mutL*::*cam* (pMN100) [expresses *dnaN-P20A*] | This work |
| MN129 | MG1655: ∆*dnaN*::(*kan*, *sacB*) ∆*mutL*::*cam* (pMN101) [expresses *dnaN-L21A*] | This work |
| MN130 | MG1655: ∆*dnaN*::(*kan*, *sacB*) ∆*mutL*::*cam* (pMN102) [expresses *dnaN-G22A*] | This work |
| MN131 | MG1655: ∆*dnaN*::(*kan*, *sacB*) ∆*mutL*::*cam* (pMN103) [expresses *dnaN-G23A*] | This work |
| MN132 | MG1655: ∆*dnaN*::(*kan*, *sacB*) ∆*mutL*::*cam* (pMN104) [expresses *dnaN-R24A*] | This work |
| MN133 | MG1655: ∆*dnaN*::(*kan*, *sacB*) ∆*mutL*::*cam* (pMN105) [expresses *dnaN-P25A*] | This work |
| MN134 | MG1655: ∆*dnaN*::(*kan*, *sacB*) ∆*mutL*::*cam* (pMN106) [expresses *dnaN-T26A*] | This work |
| MN135 | MG1655: ∆*dnaN*::(*kan*, *sacB*) ∆*mutL*::*cam* (pMN107) [expresses *dnaN-L27A*] | This work |
| MN136 | MG1655: ∆*dnaN*::(*kan*, *sacB*) ∆*mutL*::*cam* (pMN108) [expresses *dnaN-H148A*] | This work |
| MN137 | MG1655: ∆*dnaN*::(*kan*, *sacB*) ∆*mutL*::*cam* (pMN109) [expresses *dnaN-Q149A*] | This work |
| MN138 | MG1655: ∆*dnaN*::(*kan*, *sacB*) ∆*mutL*::*cam* (pMN111) [expresses *dnaN-V151A*] | This work |
| MN139 | MG1655: ∆*dnaN*::(*kan*, *sacB*) ∆*mutL*::*cam* (pMN113) [expresses *dnaN-Y153A*] | This work |
| MN140 | MG1655: ∆*dnaN*::(*kan*, *sacB*) ∆*mutL*::*cam* (pMN114) [expresses *dnaN-Y154A*] | This work |
| MN141 | MG1655: ∆*dnaN*::(*kan*, *sacB*) ∆*mutL*::*cam* (pMN115) [expresses *dnaN-K12E*] | This work |
| MN142 | MG1655: ∆*dnaN*::(*kan*, *sacB*) ∆*mutL*::*cam* (pMN116) [expresses *dnaN-Q15A*] | This work |
| MN143 | MG1655: ∆*dnaN*::(*kan*, *sacB*) ∆*mutL*::*cam* (pMN117) [expresses *dnaN-Q16A*] | This work |
| MN144 | MG1655: ∆*dnaN*::(*kan*, *sacB*) ∆*mutL*::*cam* (pMN118) [expresses *dnaN-R73A*] | This work |
| MN145 | MG1655: ∆*dnaN*::(*kan*, *sacB*) ∆*mutL*::*cam* (pMN119) [expresses *dnaN-R80A*] | This work |
| MN146 | MG1655: ∆*dnaN*::(*kan*, *sacB*) ∆*mutL*::*cam* (pMN120) [expresses *dnaN-R197A*] | This work |
| MN147 | MG1655: ∆*dnaN*::(*kan*, *sacB*) ∆*mutL*::*cam* (pMN121) [expresses *dnaN-K198E*] | This work |
| **Plasmid DNAs** | | |
| **Plasmid** | **Relevant characteristics** | **Source** |
| Zero Blunt TOPO | Kan^R^; pUC origin; commercial TOPO cloning vector | ThermoFisher |
| pDnaA-ZB | Kan^R^; Zero Blunt TOPO containing 762 bp *‘dnaA* fragment | This work |
| pKanR-PCR TOPO | Kan^R^; Zero Blunt TOPO containing 945 bp *kan* fragment | This work |
| pSacB-ZB | Kan^R^; Zero Blunt TOPO containing 1,714 bp *sacB* fragment | This work |
| pRecFZB | Kan^R^; Zero Blunt TOPO containing 700 bp *recF’* fragment | This work |
| pAK-PCR TOPO | Kan^R^; Zero Blunt TOPO containing ligated *‘dnaA*–*kan* fragment | This work |
| pSF-ZB | Kan^R^; Zero Blunt TOPO containing ligated *sacB*–*recF’* fragment | This work |
| pAKSF-PCR TOPO | Kan^R^; Zero Blunt TOPO containing ligated *‘dnaA*–*kan*–*sacB*–*recF’* fragment (i.e., *‘dnaA*–∆*dnaN*::(*kan*, *sacB*)–r*ecF’*) | This work |
| pKD46 | Amp^R^; *repA101*(Ts) pSC101 origin; expresses λRed recombinase from P*araBAD* | [[6](#_ENREF_6)] |
| pWSK29 | Amp^R^; pSC101 origin; cloning vector | [[7](#_ENREF_7)] |
| pJD100 | Amp^R^; pWSK29 expressing physiological levels of *dnaN^+^* | [[8](#_ENREF_8)] |
| pMN100 | Amp^R^; pWSK29 expressing physiological levels of *dnaN-P20A* | This work |
| pMN101 | Amp^R^; pWSK29 expressing physiological levels of *dnaN-L21A* | This work |
| pMN102 | Amp^R^; pWSK29 expressing physiological levels of *dnaN-G22A* | This work |
| pMN103 | Amp^R^; pWSK29 expressing physiological levels of *dnaN-G23A* | This work |
| pMN104 | Amp^R^; pWSK29 expressing physiological levels of *dnaN-R24A* | This work |
| pMN105 | Amp^R^; pWSK29 expressing physiological levels of *dnaN-P25A* | This work |
| pMN106 | Amp^R^; pWSK29 expressing physiological levels of *dnaN-T26A* | This work |
| pMN107 | Amp^R^; pWSK29 expressing physiological levels of *dnaN-L27A* | This work |
| pMN108 | Amp^R^; pWSK29 expressing physiological levels of *dnaN-H148A* | This work |
| pMN109 | Amp^R^; pWSK29 expressing physiological levels of *dnaN-Q149A* | This work |
| pMN110 | Amp^R^; pWSK29 expressing physiological levels of *dnaN-D150A* | This work |
| pMN111 | Amp^R^; pWSK29 expressing physiological levels of *dnaN-V151A* | This work |
| pMN112 | Amp^R^; pWSK29 expressing physiological levels of *dnaN-R152A* | This work |
| pMN113 | Amp^R^; pWSK29 expressing physiological levels of *dnaN-Y153A* | This work |
| pMN114 | Amp^R^; pWSK29 expressing physiological levels of *dnaN-Y154A* | This work |
| pMN115 | Amp^R^; pWSK29 expressing physiological levels of *dnaN-K12E* | This work |
| pMN116 | Amp^R^; pWSK29 expressing physiological levels of *dnaN-Q15A* | This work |
| pMN117 | Amp^R^; pWSK29 expressing physiological levels of *dnaN-Q16A* | This work |
| pMN118 | Amp^R^; pWSK29 expressing physiological levels of *dnaN-R73A* | This work |
| pMN119 | Amp^R^; pWSK29 expressing physiological levels of *dnaN-R80A* | This work |
| pMN120 | Amp^R^; pWSK29 expressing physiological levels of *dnaN-R197A* | This work |
| pMN121 | Amp^R^; pWSK29 expressing physiological levels of *dnaN-K198E* | This work |

**Supplementary References**

1. Blattner FR, Plunkett G, 3rd, Bloch CA, Perna NT, Burland V, et al. (1997) The complete genome sequence of *Escherichia coli* K-12. Science 277: 1453-1462.

2. Heltzel JM, Maul RW, Scouten Ponticelli SK, Sutton MD (2009) A model for DNA polymerase switching involving a single cleft and the rim of the sliding clamp. Proc Natl Acad Sci U S A 106: 12664-12669.

3. Ho C, Kulaeva OI, Levine AS, Woodgate R (1993) A rapid method for cloning mutagenic DNA repair genes: isolation of *umu*-complementing genes from multidrug resistance plasmids R391, R446b, and R471a. J Bacteriol 175: 5411-5419.

4. Fernandez De Henestrosa AR, Ogi T, Aoyagi S, Chafin D, Hayes JJ, et al. (2000) Identification of additional genes belonging to the LexA regulon in *Escherichia coli*. Mol Microbiol 35: 1560-1572.

5. Lopez de Saro FJ, Marinus MG, Modrich P, O'Donnell M (2006) The beta sliding clamp binds to multiple sites within MutL and MutS. J Biol Chem 281: 14340-14349.

6. Datsenko KA, Wanner BL (2000) One-step inactivation of chromosomal genes in *Escherichia coli* K-12 using PCR products. Proc Natl Acad Sci U S A 97: 6640-6645.

7. Wang RF, Kushner SR (1991) Construction of versatile low-copy-number vectors for cloning, sequencing and gene expression in *Escherichia coli*. Gene 100: 195-199.

8. Sutton MD (2004) The *Escherichia coli dnaN159* mutant displays altered DNA polymerase usage and chronic SOS induction. J Bacteriol 186: 6738-6748.
