# Supplementary material for: Identification of β Clamp-DNA Interaction Regions That Impair the Ability of E. coli to Tolerate Specific Classes of DNA Damage
Source: PLoS One. 2016 Sep 29;11(9):e0163643. doi: 10.1371/journal.pone.0163643 (PMC5042465; doi:10.1371/journal.pone.0163643)
Supplement: S2 Table — (DOCX) [file pone.0163643.s006.docx]

**S2 Table: Oligonucleotides used in this study.**

| **Oligonucleotide** | **Nucleotide sequence (5’-3’)** | **Source** |
| --- | --- | --- |
| DnaA-BamHI-Fwd | GGATCCAACGCTACTACCGTTCCGTAG | IDT |
| DnaA-NheI-Rev | GCTAGCAAATTTCATAGGTTTACGATGACAA | IDT |
| KanR-NheI-Fwd | GCTAGCCCGGAATTGCCAGCTGGGG | IDT |
| KanR-XhoI-Rev | CTCGAGTCAGAAGAACTCGTCAAGAAGGC | IDT |
| SacB-XhoI-Fwd | CTCGAGGGAAAATAGACCAGTTGCAATCC | IDT |
| SacB-NotI-Rev | GCGGCCGCAACCGGATATCGGCATTTTCTTTTGCG | IDT |
| RecF-NotI-Fwd | GCGGCCGCATGTCCCTCACCCGCTTGTTG | IDT |
| RecF-XbaI-Rev | TCTAGATCTGTCTCTTTCTCCCAGCCG | IDT |
| DnaAP | CATGAATGTTTCAGCCTTAGTC | IDT |
| RecF-Bottom | CAACGTTTCTCGAGCATTTATACTTGG | IDT |
| P20A Top | GGTGAGCGGTGCGTTAGGTGGTC | IDT |
| P20A Bot | GACCACCTAACGCACCGCTCACC | IDT |
| L21A Top | GAGTGGTCCGGCAGGTGGTCGTC | IDT |
| L21A Bot | GACGACCACCTGCCGGACCACTC | IDT |
| G22A Top | GGTCCGTTAGCTGGTCGTCCTAC | IDT |
| G22A Bot | GTAGGACGACCAGCTAACGGACC | IDT |
| G23A Top | GTCCGTTAGGTGCTCGTCCTACGC | IDT |
| G23A Bot | GCGTAGGACGAGCACCTAACGGAC | IDT |
| R24A Top | CGTTAGGTGGTGCTCCTACGCTAC | IDT |
| R24A Bot | GTAGCGTAGGAGCACCACCTAACG | IDT |
| P25A Top | GTTAGGTGGTCGTGCTACCCTTCCG | IDT |
| P25A Bot | CGGAAGGGTAGCACGACCACCTAAC | IDT |
| T26A Top | GTCGTCCTGCGCTACCGATTCTCG | IDT |
| T26A Bot | CGAGAATCGGTAGCGCAGGACGAC | IDT |
| L27A Top | GTCGTCCTACGGCACCGATTCTCG | IDT |
| L27A Bot | CGAGAATCGGTGCCGTAGGACGAC | IDT |
| H148A Top | CAGTTTTCTATGGCGGCTCAGGACGTTCGCTATTACTTAAATGG | Sigma-Genosys |
| H148A Bot | CCATTTAAGTAATAGCGAACGTCCTGAGCCGCCATAGAAAACTG | Sigma-Genosys |
| Q149A For | GCGAACGTCTGCATGCGCCATAGAAAACTGGG | Bio Basic |
| Q149A Rev | CCCAGTTTTCTATGGCGCATGCAGACGTTCGC | Bio Basic |
| D150A For | CCATTTAAGTAATAGCGAACGGCCTGATGCGC | Bio Basic |
| D150A Rev | GCGCATCAGGCCGTTCGCTATTACTTAAATGG | Bio Basic |
| V151A For | CCATTTAAGTAATAGCGAGCGTCCTGATGCG | Bio Basic |
| V151A Rev | CGCATCAGGACGCTCGCTATTACTTAAATGG | Bio Basic |
| R152A Top | CAGTTTTCTATGGCGCATCAGGACGTTGCCTATTACTTAAATGG | Sigma-Genosys |
| R152A Bot | CCATTTAAGTAATAGGCAACGTCCTGATGCGCCATAGAAAACTG | Sigma-Genosys |
| Y153A For | CCATTTAAGTAAGCGCGAACGTCCTGATGCG | Bio Basic |
| Y153A Rev | CGCATCAGGACGTTCGCGCTTACTTAAATGG | Bio Basic |
| Y154A For | GCATACCATTTAAGGCATAGCGAACGTCC | Bio Basic |
| Y154A Rev | GGACGTTCGCTATGCCTTAAATGGTATGC | Bio Basic |
| K12E Top | GCGGTTCTAATAAATGCTCACG | IDT |
| K12E Bot | CGTGAGCATTTATTAGAACCGC | IDT |
| Q15A Top | GGACCGCTCACCTGTGCTAGCGG | IDT |
| Q15A Bot | CCGCTAGCACAGGTGAGCGGTCC | IDT |
| Q16A Top | GGACCGCTCACCGCTTGTAGCG | IDT |
| Q16A Bot | CGCTACAAGCGGTGAGCGGTCC | IDT |
| R73A Top | GCAGATATCAAAGAATTTGGCCGCCG | IDT |
| R73A Bot | CGGCGGCCAAATTCTTTGATATCTGC | IDT |
| R80A Top | GGCAGACCAGCGCAGATATCAAAG | IDT |
| R80A Bot | CTTTGATATCTGCGCTGGTCTGCC | IDT |
| R197A Top | CACGCCTTTAGCCGGTACGATCACC | IDT |
| R197A Bot | GGTGATCGTACCGGCTAAAGGCGTG | IDT |
| K198E Top | CACGCCTTCACGCGGTACGATCACC | IDT |
| K198E Bot | GGTGATCGTACCGCGTGAAGGCGTG | IDT |
| Beta For1 SP | GACGTTCGCTATTACTTAAATGG | Sigma-Genosys |
| Beta Rev2 SP | GCCAATCTTGTACGCGCAG | Sigma-Genosys |
| oriC-1 | CTGTGAATGATCGGTGATCC | IDT |
| oriC-2 | AGCTCAAACGCATCTTCCAG | IDT |
| terC-1 | CAGAGCGATATATCACAGCG | IDT |
| terC-2 | TATCTTCCTGCTCAACGGTC | IDT |
